# Inhibiting insulin and mTOR signaling by afatinib and crizotinib combination fosters broad cytotoxic effects in cutaneous malignant melanoma

**DOI:** 10.1038/s41419-020-03097-2

**Published:** 2020-10-20

**Authors:** Ishani Das, Huiqin Chen, Gianluca Maddalo, Rainer Tuominen, Vito W. Rebecca, Meenhard Herlyn, Johan Hansson, Michael A. Davies, Suzanne Egyházi Brage

**Affiliations:** 1 Department of Oncology-Pathology, Karolinska Institutet, 171 64 Stockholm, Sweden; 2 Department of Biostatistics, The University of Texas MD Anderson Cancer Center, Houston, TX USA; 3 Science for Life Laboratory, School of Biotechnology, KTH Royal Institute of Technology, Stockholm, Sweden; 4 Molecular and Cellular Oncogenesis, The Wistar Institute, Philadelphia, PA 19104 USA; 5 Department of Melanoma Medical Oncology, Division of Cancer Medicine, The University of Texas MD Anderson Cancer Center, Houston, TX USA

**Keywords:** Cancer therapeutic resistance, Targeted therapies, Melanoma, Tumour biomarkers

## Abstract

Current treatment modalities for disseminated cutaneous malignant melanoma (CMM) improve survival, however disease progression commonly ensues. In a previous study we identified afatinib and crizotinib in combination as a novel potential therapy for CMM independent of *BRAF/NRAS* mutation status. Herein, we elucidate the underlying mechanisms of the combination treatment effect to find biomarkers and novel targets for development of therapy that may provide clinical benefit by proteomic analysis of CMM cell lines and xenografts using mass spectrometry based analysis and reverse phase protein array. Identified candidates were validated using immunoblotting or immunofluorescence. Our analysis revealed that mTOR/Insulin signaling pathways were significantly decreased by the afatinib and crizotinib combination treatment. Both in vitro and in vivo analyses showed that the combination treatment downregulated pRPS6KB1 and pRPS6, downstream of mTOR signaling, and IRS-1 in the insulin signaling pathway, specifically ablating IRS-1 nuclear signal. Silencing of RPS6 and IRS-1 alone had a similar effect on cell death, which was further induced when IRS-1 and RPS6 were concomitantly silenced in the CMM cell lines. Silencing of IRS-1 and RPS6 resulted in reduced sensitivity towards combination treatment. Additionally, we found that IRS-1 and RPS6KB1 expression levels were increased in advanced stages of CMM clinical samples. We could demonstrate that induced resistance towards combination treatment was reversible by a drug holiday. CD171/L1CAM, mTOR and PI3K-p85 were induced in the combination resistant cells whereas AXL and EPHA2, previously identified mediators of resistance to MAPK inhibitor therapy in CMM were downregulated. We also found that CD171/L1CAM and mTOR were increased at progression in tumor biopsies from two matched cases of patients receiving targeted therapy with BRAFi. Overall, these findings provide insights into the molecular mechanisms behind the afatinib and crizotinib combination treatment effect and leverages a platform for discovering novel biomarkers and therapy regimes for CMM treatment.

## Introduction

The availability of targeted therapy and immunotherapy has revolutionized the treatment of cutaneous malignant melanoma (CMM) patients with advanced disease. The use of targeted therapies, comprised of a combination of BRAF and MEK inhibitors (targeting the mitogen activated protein kinase (MAPK) signaling pathway), being limited to patients harboring *BRAF* mutant CMM, leaves patients with *BRAF* WT with fewer treatment options. Those who initially respond to either immunotherapy or targeted therapy often develop acquired resistance associated with a similar 5 years overall survival (OS) rate for both treatment modalities of 34–52%^1–3^. Different factors have been attributed towards MAPK inhibitor (MAPKi) resistance^4,5^. One of them is over-expression of receptor tyrosine kinases (RTKs) including AXL, MET, EGFR, ERBB3, IGF1R that lead to reactivation of the MAPK and/or PI3K-AKT pathways^6^. However, it has been demonstrated that resistance can be overcome by inhibiting RTKs in MAPKi resistant CMM cells^7–9^.

Crosstalk between the tumor microenvironment and cancer cells and within tumor cells themselves prime them to adopt aberrations or to switch to compensatory regulatory pathways to override the effects of a single therapeutic agent. Recent studies have highlighted the need of using combinatorial strategies to block multiple targets simultaneously^2,10,11^. Crosstalk between different RTKs could mediate resistance to single treatment with RTK inhibitor. MET and IGF1R have been posited to contribute to resistance to EGFR inhibitor in lung cancer^12,13^. Furthermore, resistance to ERBB family inhibitor afatinib could be overcome by combining afatinib with the MET/ALK inhibitor crizotinib^14^. Therefore, discovering new biomarkers and combining different therapeutic strategies underscores the need for future developments of CMM therapy. We have recently shown that knockdown of MET caused an up-regulation of EGFR and induction of pAKT in BRAF inhibitor resistant CMM cells, which was blocked by simultaneous knockdown of EGFR and MET. Furthermore, we have shown that combining afatinib and crizotinib achieves an additive/synergistic effect in CMM cells and xenograft, independent of *BRAF/NRAS* mutation status^8^.

Herein we sought to unravel molecular mechanisms behind the effect of the novel afatinib and crizotinib combination therapy by proteomic analysis of a panel of CMM cell lines and xenografts using mass spectrometry and RPPA. The candidate proteins chosen for further validation as targets of the combination treatment were based on the above findings and additionally validated by immunoblotting and immunofluorescence. The candidates were also related to *BRAF/NRAS* mutational status and tumor stage in clinical samples. Furthermore, we induced resistance towards the combination treatment in CMM cells to identify potential candidates involved in resistance and to analyze whether resistance is reversible by discontinuing the treatment.

## Material and methods

### Xenograft and inhibitors

A375 cells (3.6 × 10^6^) were mixed 1:1 with growth factor reduced matrigel matrix (VWR) and injected subcutaneously in the flank of 6 week old CB-17/Icr-*Prkdc*^*scid/scid*^ female mice (Janvier)^8^.

Afatinib and crizotinib were purchased from Selleck Chem (USA). For all in vitro treatments, the compounds were dissolved in DMSO.

For additional information, see Supplementary Material and Methods.

## Results

### High throughput proteomic analysis implicates proteins involved in mTOR and insulin signaling pathways upon combinatorial treatment in CMM cells

**Fig. 1:**
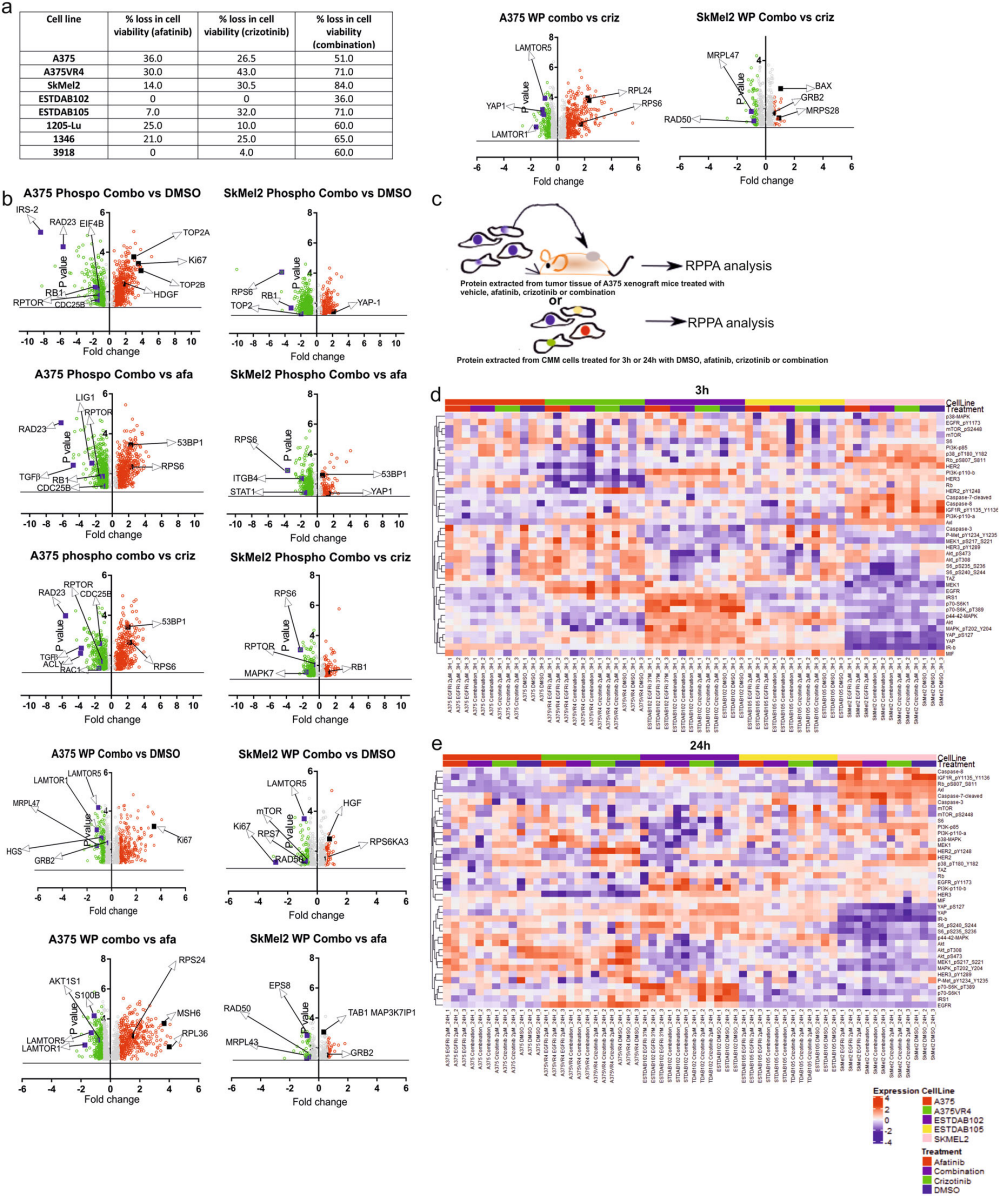
Combination deregulates mTOR and Insulin pathways in CMM cells. **a** Table showing loss in cell viability after single or combination treatment in 5 CMM cell lines (data presented as mean; *n* = 3 technical replicates; *p* < 0.001). **b** Volcano plots showing proteins altered in A375 (*BRAF mutant*) and SKMel2 (*NRAS mutant*) as assessed by LC MS-based phosphoproteomics or whole proteomics (WP) comparing combination treatment with DMSO, afatinib or crizotinib. Linear cut off *p* < 0.05 and 1.5 fold change (*n* = 3 technical replicates) was used to identify differentially expressed proteins, up-regulated (red) and down-regulated (green) proteins. Here we found that in phosphoproteomics the number of significantly differentially expressed proteins ranged between 300–1100 in the different treatment comparisons, whereas in WP it was around 400–1360 proteins. **c** Illustration of CMM cells and protein lysates extraction from A375 xenograft tumor tissue for RPPA. **d** Heat map with supervised clustering showing changes in selected candidate proteins analyzed by RPPA after 3 h or **e** 24 h of treatment with DMSO, single or combination drugs (*n* = 3).

We have recently described that CMM cells are sensitive to the combination treatment of afatinib and crizotinib independent of *BRAF/NRAS* mutational status and in this follow up study, three additional cell lines, 1205-Lu, 1346 and 3918 were included to validate this finding^8^ (Fig. 1a, Supplementary Fig. S1a). To assess drug toxicity on normal cells, we treated human dermal fibroblasts and human normal epithelial keratinocytes. Both cells showed a modest (~20–30%) reduction in cell viability upon combination treatment (Supplementary Fig. S1b). We employed total and phosphorylated proteomics platforms to elucidate the underlying mechanisms of the combinatorial effect of afatinib and crizotinib treatment. In this respect, we compared early proteomic response upon 3 h exposure to afatinib, crizotinib and thereof combination in two CMM cell lines, *BRAF* mutated A375 and *NRAS* mutated SKMel2. Overall, ~4500 proteins were detected. The principal component analysis (PCA) showed a distinct separation between the two cell lines (Supplementary Fig. S2a). Pairwise comparison of the combinatorial drug treatment versus the control (DMSO) and single treatment was performed to identify significantly differentially expressed proteins (linear *p* < 0.05, linear fold change = 1.5), with a false discovery rate of 1% and plotted as volcano plots and venn diagrams (Fig. 1b, Supplementary Fig. S2b). Approximately 31–35% of these proteins were differentially altered in all three paired comparison groups in A375, whereas only approximately 1–15% in SkMel2, both regarding total and phosphoproteome analysis (Supplementary Fig. S2b).

Analysis of biological processes associated with the altered top 200 up or down-regulated phosphorylated or total proteins common in at least two out of three control/treatment groups compared to the combination treatment group in both cell lines included RNA splicing, ribonucleoprotein complex assembly, DNA repair and apoptosis. (Supplementary Fig. S3). KEGG pathway analysis using the same set of altered proteins showed that the combination treatment group had target hits belonging to the mTOR, insulin signaling, DNA damage response, ribosome processing and JAK-STAT signaling pathways (Supplementary Table 1a–d).

In both cell lines, we observed that the afatinib and crizotinib combination treatment in comparison to single treatments or DMSO caused a significant down-regulation of proteins (Fig. 1b) belonging to the Ragulator complex which has been shown to display a multifaceted role in lysosomal signaling and trafficking^15^ and also been implicated in controlling mTORC1 and ERK signaling^16^. In addition, we found AKTS1 to be down-regulated in A375 which supports our recent observation in our xenograft model^8^, whereas pMAPK7 was decreased in SKMel2 (Fig. 1b).

### RPS6KB1, RPS6 and IRS-1 are downregulated by the combination treatment in *vitro*

We performed RPPA to capture both early (3 h) and later (24 h) alterations induced by afatinib and crizotinib alone or in combination in A375, A375VR4, SkMel2, ESTDAB102 and ESTDAB105 cell lines. When comparing the list of altered proteins and heatmaps with supervised clustering presented for selected proteins at 3 h and 24 h post treatment, we found that pRPS6 was significantly inhibited after 3 h afatinib and crizotinib combination treatment in all five cell lines (Fig. 1d, e, Supplementary Fig. S4). We were also able to confirm that pAKT was down-regulated in all cell lines after 3 h in comparison to the other treatment arms, thus corroborating our MS data where we observed down-regulation of the AKT substrate AKT1S1 (Fig. 1d, e, Supplementary Fig. S4). pAKT still remained downregulated after 24 h treatment in three of the cell lines. In addition, these data confirm our observation in a recent study showing downregulation of pAKT in vivo after afatinib and crizotinib combination treatment^8^. IRS-2 in the insulin signaling was among the top down-regulated candidates in A375 phosphoproteomic analysis when comparing DMSO vs combo. However, since this protein was absent in the RPPA panel, we checked for another insulin receptor substrate, IRS-1, instead and observed a slight overall decrease of total IRS-1 in protein expression at 24 h post combination treatment in SkMel2 and A375VR4 (Fig. 1e).

### Combination treatment downregulates key players in the mTOR and insulin signaling pathways in a xenograft model

**Fig. 2:**
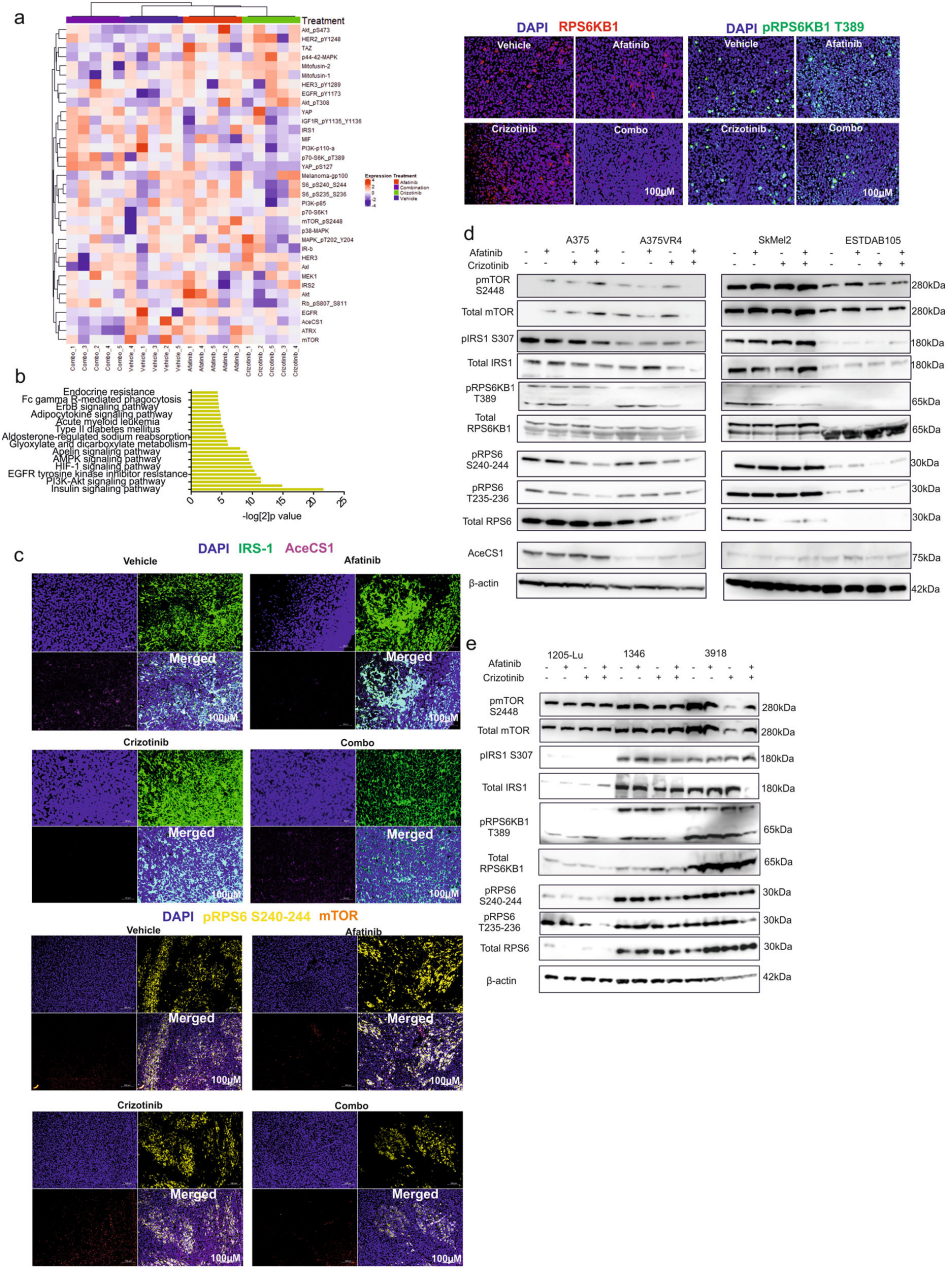
Afatinib and crizotinib in combination downregulates IRS-1, RPS6KB1 and RPS6 *in vivo and in vitro*. **a** Heat map with supervised clustering showing protein expression changes in selected candidates analyzed by RPPA using tumor tissue lysates (*n* = 20; 5 per group) from A375 xenograft mice treated with vehicle, afatinib and crizotinib alone and in combination for 14 days (5 consecutive days, followed by two days drug off and repetition of same regimen for one more week). **b** KEGG pathway analysis of candidates significantly altered in **a**. **c** Validation of IRS-1, AceCS1, pRPS6, pRPS6KB1 and total RPS6KB1 in xenograft tumor tissues using IF. **d**, **e** In vitro validation of selected candidate proteins after 3 h treatment with DMSO, afatinib and crizotinib alone and in combination in seven CMM cell lines (*n* = 2).

We further investigated if alterations in the same pathways and proteins were associated with the combination treatment effect also in an in vivo model. Proteins were isolated from vehicle, afatinib, crizotinib and combination treated tumor xenografts (established by injecting A375 cells) as described in^8^ and assessed utilizing RPPA (Fig. 1c). Our overall results showed that proteins found to be deregulated by the afatinib and crizotinib combination treatment belong to the insulin and PI3K-AKT signaling pathways (Fig. 2a, b, Supplementary Fig. S5a). Since the mTOR pathway in conjunction with downstream effector partners has been shown to modulate the signaling of IRS-1 through a negative feedback loop in a cell autonomous fashion^17^, and our pathway analysis also indicated that cellular pathways connected to mTOR and insulin were de-regulated, we decided to focus on the mTOR and the insulin signaling pathways. Due to the high false discovery rate (FDR) in RPPA analysis of the xenograft data, we validated our results using IF for mTOR, IRS-1, AceCS1, pRPS6KB1 and total RPS6KB1. We also included pRPS6 since it was downregulated in all cell lines after afatinib and crizotinib combination treatment and is downstream of RPS6KB1.Three xenograft samples from each treatment group were randomly selected for this. We found a decrease in expression of pRPS6, pRPS6KB1 (as indicated by the reduced number of pRPS6KB1 positive cells; *p* < 0.0001) (Supplementary Fig. S5b) and total RPS6KB1, but could not observe any down-regulation of mTOR and AceCS1 by the afatinib and crizotinib combination treatment when compared to single agent treatments or vehicle (Fig. 2c). Interestingly, an overall decrease in IRS-1 expression which has previously been demonstrated to mediate BRAFi resistance was found^18^ (Fig. 2c). Moreover, in two of three validated cases we observed that tumor cells in the combination treatment group lacked nuclear IRS-1 staining while all other treatment arms had discernable nuclear accumulation of IRS-1 (Fig. 2c).

To further validate the MS and RPPA candidates we treated seven CMM cell lines with afatinib and crizotinib alone or in combination for 3 h and analyzed alterations in protein expression by immunoblotting. We were able to confirm that IRS-1 was reduced after combination treatment, either by down-regulation of total IRS-1 in A375, A375VR4 and 3918, or by induction of the inactive form pIRS-1 S307 in 3918 and SkMEl2 (Fig. 2d, e). We also observed a consistent down-regulation of p-RPS6KB1 and RPS6 (5/6) excluding ESTDAB105 which had very low intrinsic levels of these two proteins after afatinib and crizotinib combination treatment. We only observed a down-regulation of mTOR in A375VR4 (Fig. 2c–e). Acetyl-CoA is involved in metabolism, and increasing evidence suggests the importance of addiction of cancer cells to carbon metabolism^19^. According to our RPPA results, AceCS1, an Acetyl-CoA synthetase enzyme was significantly down-regulated by the combination treatment. However, we were unable to verify this using both IF and immunoblotting (Fig. 2c, d).

### Co-silencing of IRS-1 and RPS6 further induces apoptosis than silencing each gene alone in CMM cells

**Fig. 3:**
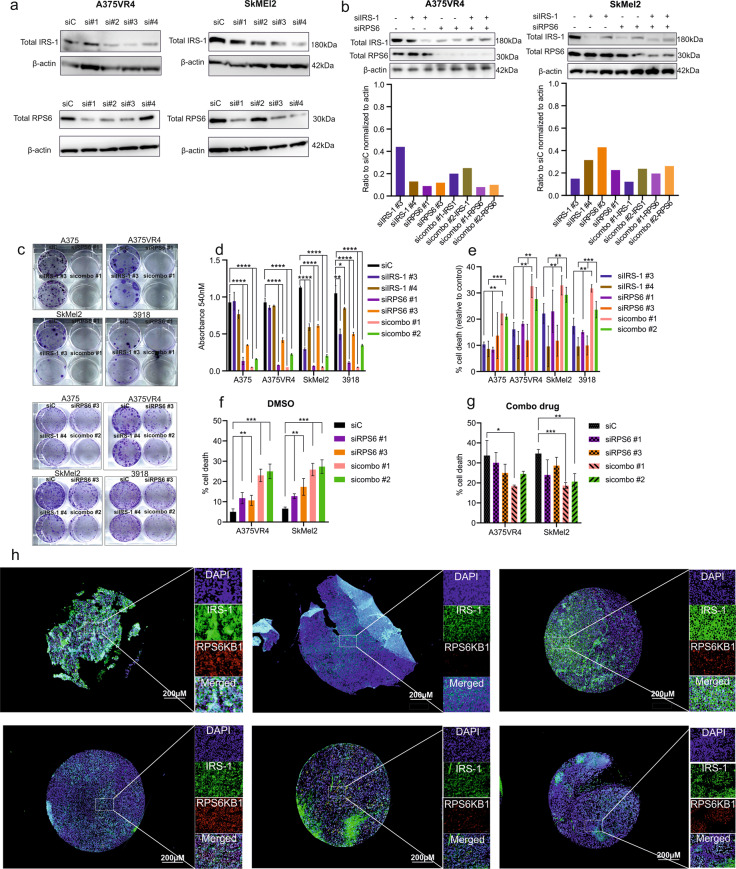
Co-silencing IRS-1 and RPS6 potentiates apoptotic cell death and ablates cellular proliferation. **a** Immunoblotting showing efficient knockdown of siRNAs against IRS-1 and RPS6 used in this study. **b** Immunoblotting showing IRS-1 siRNA (#3, #4) and RPS6 siRNA (#1, #3) in single and combination knockdowns. These siRNAs were chosen for further validation studies. In all subsequent experiments sicombo #1 (siIRS-1 #3 + siRPS6 #1) and sicombo#2 (siIRS-1 #4 + siRPS6 #3). **c** Colony formation assay showing reduced proliferation after knocking down RPS6 alone or in combination with IRS-1. **d** Quantification of **c** (error bars represent mean ± SD; *n* = 3). **e** FACS analysis quantification showing concomitant silencing of RPS6 and IRS-1 for 48 h further induces apoptotic cell death than silencing either gene alone (error bars represent mean ± SD; *n* = 3). **f**, **g** FACS analysis quantification showing CMM cells lose sensitivity towards combination drug treatment upon concomitant silencing of RPS6 and IRS-1. Silencing was performed for 24 h followed by 24 h drug treatment with afatinib 2 µM + crizotinib 2 µM. **h** Protein expression and localization patterns of IRS-1 and RPS6KB1 in a cohort of tumor samples (*n* = 60) from CMM patients. Boxed area are enlarged to show more detailed staining of IRS-1 and RPS6KB1.

IRS-1 and RPS6, downstream of RPS6KB1, were downregulated after combination treatment and overexpression of IRS-1 and RPS6 have both been implicated in development of resistance towards MAPKi^18,20^. We therefore investigated what impact these two genes have on cellular proliferation and cell death by silencing them alone or co-silencing. We selected four cell lines (A375, A375VR4, SkMel2 and 3918) based on the oncogenic driver mutations and verified IRS-1 and RPS6 silencing using four different siRNAs (Fig. 3a). Moreover, we validated the silencing efficiencies upon silencing both IRS-1 and RPS6 for the two most promising siRNAs (Fig. 3b). While RPS6 silencing alone or combined with IRS-1 co-silencing efficiently abrogated cellular proliferation in all cell lines studied, silencing of IRS-1 caused most prominent reduction in colonies in *BRAF* wildtype cells, *NRAS* mutant SKMel2 and *BRAF/NRAS* WT 3918 (Fig. 3c, d). However, analysis by FACS showed that silencing of each gene alone had similar effect on cell death and concomitant silencing of IRS-1 and RPS6 significantly further induced cell death (Fig. 3e). To investigate if sensitivity towards the combination was altered upon silencing IRS-1 and RPS6, we performed a FACS analysis. Knockdown ensued by drug treatment for 24 h showed that atleast with sicombo #1, there was ~ 45% reduction (*p* < 0.05) in combination mediated cell death in both A375VR4 and SkMel2. Silencing of RPS6 alone did not mediate significant loss in cell death (Fig. 3f, g).

We stained for IRS-1 and RPS6KB1 total protein expression using immunofluorescence in an independent cohort of 65 stage III/IV tumors from 56 CMM patients with a subgroup receiving targeted, immunotherapy and/or chemotherapy before taking the biopsy (Fig. 3h, Supplementary Fig. S6, Supplementary Table 2). Thirteen percent (8/60, 5 were not evaluable) of the tumors had predominantly membranous IRS-1 staining, while 28% (17/60) of the samples showed mainly nuclear localization of IRS-1. Approximately 17% (10/60) of the tumors had low expression of IRS-1. Although RPS6KB1was in most cases mainly localized in the nucleus, a distinct cytoplasmic localization was also observed (Supplementary Fig. S6, Fig. 3h). In approximately 27% (16/60) of the tumors, absent-low expression of RPS6KB1 was observed of which 9 cases also had low IRS-1 expression. When comparing patients who were treatment naïve before taking the biopsy and those who received targeted therapy prior to biopsy, 21% (5/24) and 13% (2/15) had low IRS-1 expression. Fifty% IRS-1 nuclear and 94% RPS6KB1 nuclear +cytoplasmic localization. For patients who received targeted therapy prior to biopsy IRS-1 nuclear localization was ~33% and RPS6KB1 nuclear + cytoplasmic localization was ~60% (Supplementary Table 2).

### Differential protein expression and localization patterns of IRS-1, RPS6KB1 and RPS6 is observed between different stages of CMM

**Fig. 4:**
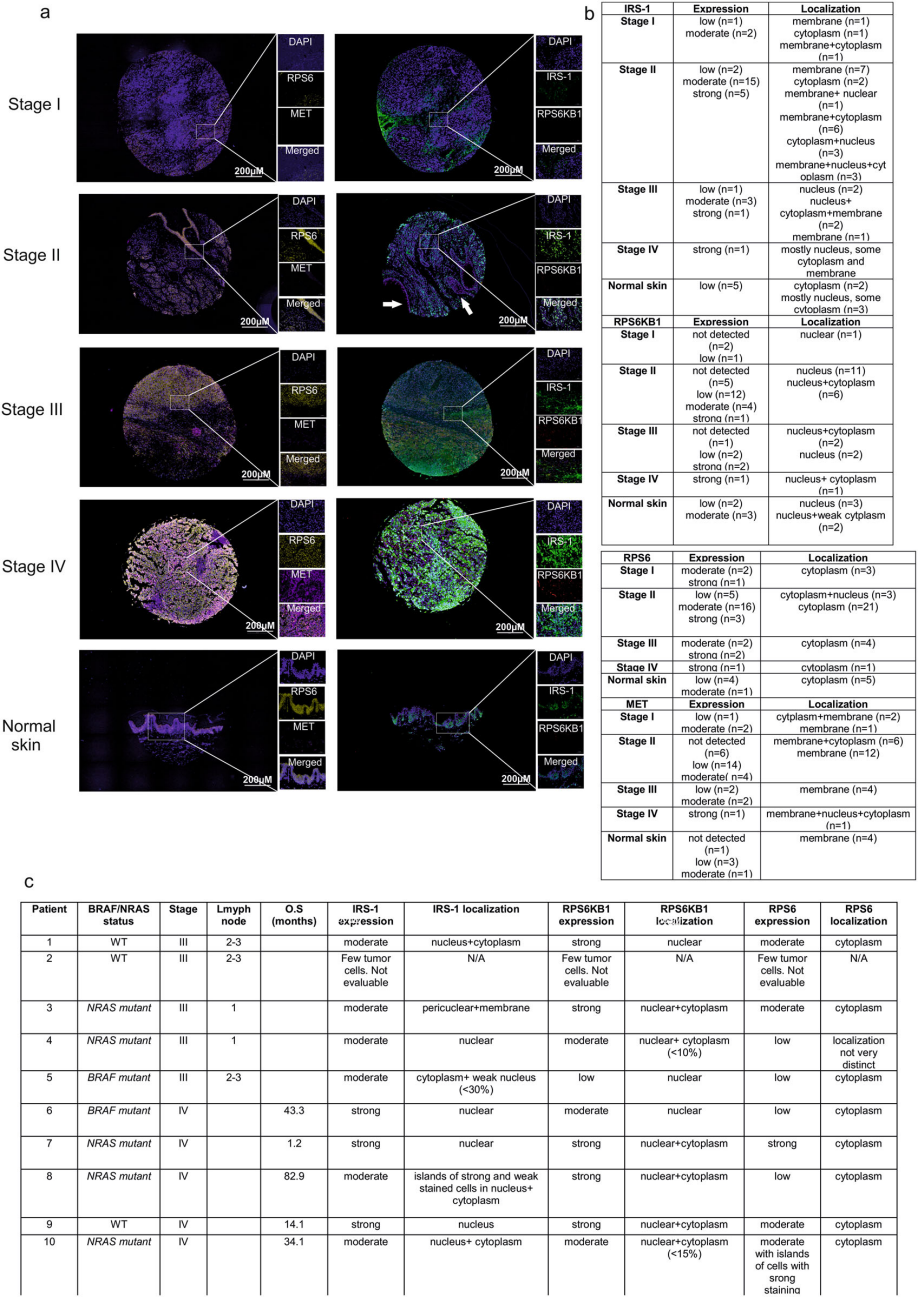
Protein expression patterns of IRS-1, RPS6KB1 and RPS6 varies with disease stage. **a** Representative samples (one from each stage I–IV and normal tissue) from TMA (Me 1002a) showing expression and localization pattern of IRS-1, RPS6KB1, RPS6 and MET. Boxed area are enlarged to show more detailed staining of IRS-1,RPS6KB1, RPS6 and MET. **b** Overall summary Table for all samples included in TMA used in **a**. **c** Summary Table of IRS-1, RPS6KB1 and RPS6 staining from nine additional CMM Stage III/IV tumor samples.

Previous studies have elucidated that RPS6KB1, RPS6 and insulin signaling pathways confer important roles in promoting CMM progression. To ascertain if the expression levels of RPS6KB1, RPS6 and IRS-1 proteins varied based on stage of CMM, we used 42 tumor samples (stage I–IV) and 5 normal skin tissues. One tumor sample was not evaluable. Our results indicate that RPS6KB1 and IRS-1 proteins have an increase in expression in advanced disease (stage III, IV) vs local disease (stage I, II) or normal skin (Fig. 4a). Approximately 53% (8/15) of stage III/IV tumors express strong RPS6KB1 compared to 4% (1/25) of stage I/II cases (Fig. 4b, c). We found nuclear localization of RPS6KB1 in all cases, interspersed with cytoplasmic localization mainly in stage IV disease. It was striking that the localization of IRS-1 was predominantly nuclear (> 70% cells expressing IRS-1 in nucleus) in 11/15 (73%) of Stage III- IV tumors but only in 28% (7/25) of the stage I-II tumors (Fig. 4b). RPS6 expression, on the contrary did not show any altered expression dependent on disease stage (Fig. 4c). Since MET has been previously described by us and others to be a contributing factor towards increased tumor aggressiveness and drug resistance and is a target of crizotinib^8,21^, we also assessed its expression in this tumor cohort. Our results indicated that MET expression was increased in stage III–IV disease (Fig. 4a, b).

### *NRAS mutant* CMMs have increased mRNA and protein levels of IRS-1 compared to *BRAF mutant* CMMs

To determine whether IRS-1, RPS6KB1 and RPS6 expression were associated with *BRAF/NRAS* driver mutation status in CMM, we analyzed data from The Cancer Genome Atlas (TCGA)^22,23^. Our results showed that IRS-1 mRNA expression was significantly (*p* < 0.01) higher among patients with *NRAS* mutant CMM vs *BRAF* mutant or *BRAF/NRAS* WT. IRS-1 remained significantly higher in *NRAS* mutant vs *BRAF* mutant melanomas at the protein level (Supplementary Fig. S7). This was also observed when comparing the DMSO control for the BRAF mutant cells with the NRAS mutant cells (Fig. 2d, e). Both RPS6 mRNA and protein expression was significantly higher in WT CMM tumors vs *BRAF* mutant (*p* < 0.05). In contrast, no difference was observed for RPS6KB1 at the mRNA level, while there was a significantly higher protein expression in WT compared to *BRAF* mutant CMMs (Supplementary Fig. S7). A similar difference was also found in the cell lines (Fig. 2d, e).

### Induced resistance towards the combination treatment is reversible after ‘drug holiday’

**Fig. 5:**
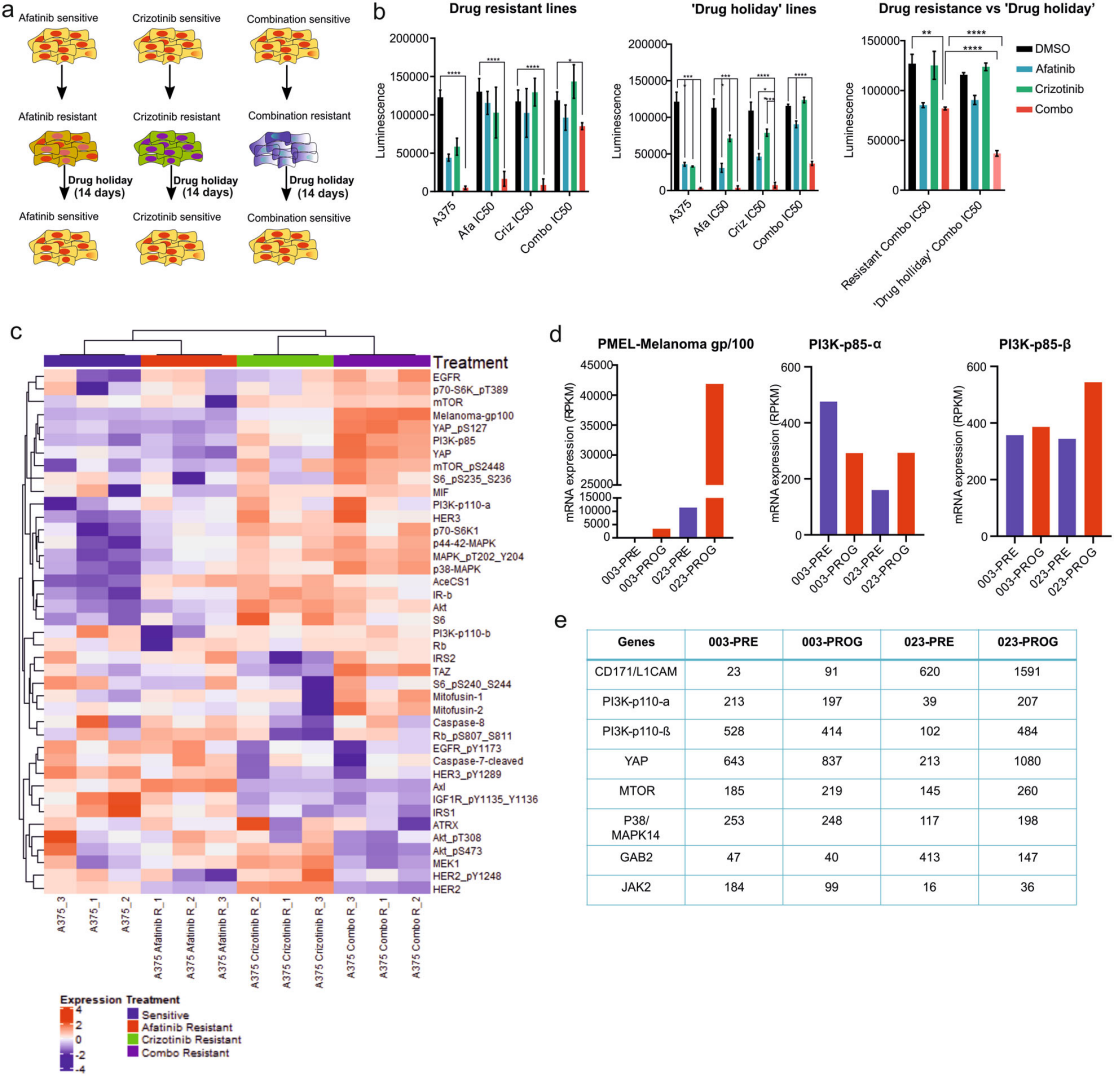
Induced resistance against afatinib and crizotinib combination is reversible. PMEL and PI3K-p85 protein expression is upregulated in afatinib and crizotinib combination resistant cells. **a** Schematic showing the ‘drug holiday’ model used in this study where A375 cells were induced with resistance towards IC50 values of afatinib, crizotinib or the combination. **b** 2D MTS assay showing cell viability in the resistant cell lines against single or combination treatment. Induced resistance could be reverted back after keeping cells off drugs for 10 days. The resistance developed to the combination treatment was more than that achieved towards either drug alone (error bars represent mean ± SD; *n* = 3; *p* < 0.01). **c** Heat map with supervised clustering showing changes in protein candidates of selected genes as analyzed by RPPA when comparing drug resistant A375 cell lines with sensitive A375 cells (*n* = 3 technical replicates). **d** mRNA expression of genes associated with afatinib and crizotinib combination resistance, PMEL and PI3K-p85, in two matched cases from CMM patients who received BRAFi, utilizing AmpliSEQ data. Samples were collected before start of therapy and at progression. **e** Table showing AmpliSEQ data from the same matched cases as in **d** for other candidates associated with afatinib and crizotinib combination resistance.

Since A375 was used for the xenograft study, we chose this cell line for induction of drug resistance towards afatinib and crizotinib alone or in combination (Fig. 5a). Cells were exposed to IC50 drug concentrations for around 2 months until the cells became resistant to IC50 doses for the drugs (Fig. 5b, Supplementary Fig. S8a). To validate if the induced resistance could be reverted, cells were subjected to a ‘drug holiday’ period for 14 days, after which they were treated again with the drugs to test for sensitivity. Our data demonstrate that induced resistance to the combination treatment is reversible (Fig. 5b). However, the ‘drug holiday’ did not alter the sensitivity to single agent therapy. RPPA was conducted to discern molecular changes that may have occurred when resistance was developed (Fig. 5c). Expression levels of many proteins were altered with resistance development including gp100/PMEL, CD171/L1CAM, GAB2, mTOR and PI3K-p85 which have been previously shown to be upregulated in tumors including CMM and promoting tumor progression^24–28^ (Fig. 5c, Supplementary Fig. S8b). Interestingly, RTKs previously associated with BRAFi resistance like AXL and EPHA2 were downregulated in the combination resistant cells^7,29^ (Supplementary Fig. S8b). We also found induction of PMEL, CD171/L1 and mTOR at progression in tumor biopsies from two matched cases of patients receiving targeted therapy with BRAFi (Fig. 5d, e). We validated our candidates associated with resistance using an external selected cohort of 10 patients with matched subcutaneous tumor samples obtained before start of treatment with BRAFi and at progression^30^. For a majority of these cases, induced expression of these resistance factors was observed at progression to a varying extent, except for PMEL/gp100 (here indicated by ratio of prog/pre >1) (Supplementary Fig. S8c).

## Discussion

**Fig. 6:**
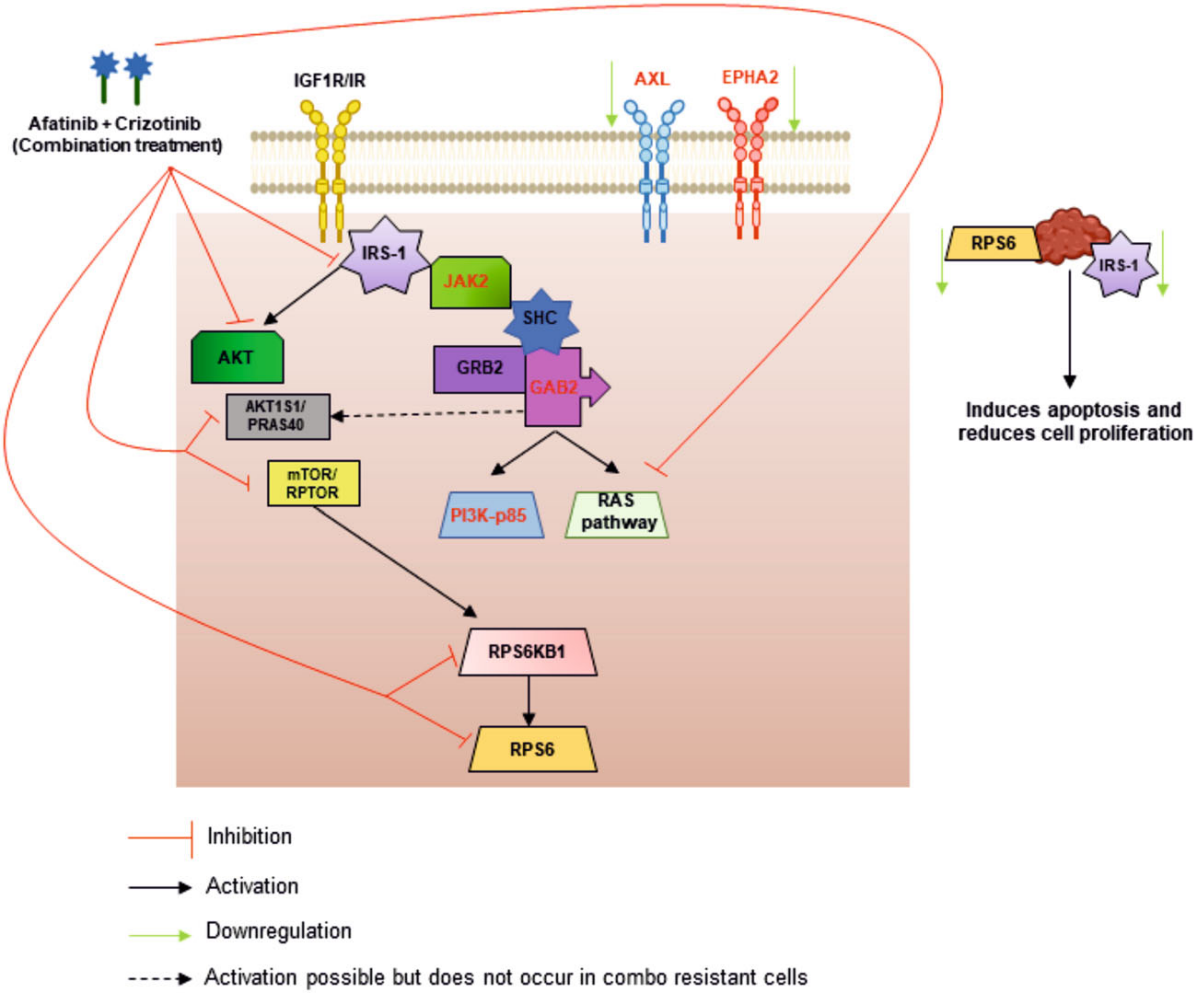
Schematic model showing proposed mechanism of action of afatinib and crizotinib combination treatment. Combination treatment with afatinib and crizotinib blocks IRS-1and downstream effector pathways like AKT and RAS. Downstream mediators of the mTOR pathway- RPS6KB1 and RPS6 are also inhibited by the combination. Co-silencing of IRS-1 and RPS6 potentiates apoptotic cell death and ablates cellular proliferation. RTKs previously associated with BRAFi resistance like AXL and EPHA2 are downregulated in the combination resistance cell line, whereas the catalytic subunit of PI3K (p85) is upregulated.

Afatinib and crizotinib have been clinically approved for non-small cell lung cancer (NSCLC), but not for CMM. In this study we have focused on investigating the molecular mechanisms underlying the broad cytotoxic effects achieved by combining afatinib and crizotinib in CMM^8^. By employing multiple proteomic approaches we demonstrate that the combination of afatinib and crizotinib mediates de-regulation of the mTOR and insulin signaling pathways (Fig. 6).

The mTOR pathway has been implicated in the emergence of resistance to BRAFi in various cancer types including CMM^31,32^. mTOR mediated resistance can occur in a cell-autonomous manner through the MAPK pathway or in a non-cell autonomous manner by dynamic rewiring of molecular signals that cause emergence of resistance to proximal cancer cells^33^. To overcome the cross-resistance to BRAFi, some studies have proposed a combination therapy utilizing inhibitors of mTOR and BRAF^34,35^. Previous studies have also highlighted activation of the mTOR pathway in tumor cells resistant to MET TKIs^28,36^.

Growing evidence suggests that RPS6KB1 and RPS6, two key proteins in the mTOR pathway, as crucial drivers of tumor onset, progression and BRAFi resistance^20,37,38^. The combination treatment significantly reduced the levels of these activated proteins in our xenograft samples. Our MS analysis highlighted that several proteins forming integral parts of ribosomes (40S and 60S subunits) were differentially deregulated. This could be an important finding, since it is well known that cancer cell survival heavily relies on protein synthesis and targeting certain ribosomal subunits inhibits melanoma development mediated through the MDM2-p53 pathway^39^.

Increasing evidence suggests that mTOR is able to phosphorylate several residues of IRS proteins in the insulin signaling pathway which affect their signaling^40^. IRS-1 has been shown to play a pivotal role in cancer cell proliferation and BRAFi therapy resistance and upregulation is observed in many malignancies^18,41^. Here we have shown that concomitant silencing of RPS6 and IRS-1 was able to further potentiate apoptotic cell death in CMM cells and cause a loss in cellular proliferation.

Predominant nuclear localization with strong IRS-1 expression was more commonly observed in stage III/IV tumors compared to stage I/II CMM tumors suggesting that nuclear IRS-1 may have an adverse effect. Interaction of nuclear IRS1 proteins with upstream binding factor 1 has been shown to cause significant activation of the ribosomal DNA promoter leading to increased rRNA synthesis^42^. Therefore, down-regulation of nuclear IRS-1 by the combination treatment of afatinib and crizotinib, as demonstrated in this study, could be clinically relevant.

In this study we have shown that resistance generated against the afatinib and crizotinib combination treatment is reversible by a ‘drug holiday’. This is in line with previous observations where it has been shown that intermittent dosing of BRAFi forestalls the onset of drug resistance^43^. gp100 (PMEL) and PI3K-p85 were among the proteins that were most induced in the cell line resistant to the combination treatment, compared to sensitive or single agent-resistant cell lines (Fig. 5). Neither of these proteins have previously been directly associated with afatinib or crizotinib resistance, however, both have been shown to be up-regulated in CMM^27,44^. Studies indicate that tumors having increased PMEL are more RAF-inhibitor sensitive^45^. Therefore, we speculate that induced PMEL expression in prospect to combination therapy resistance may make the *BRAF* mutant tumors sensitive to BRAFi/MEKi. Moreover, in neuroblastoma it has been shown that combination treatment with crizotinib and mTORC1 inhibitor led to a reciprocal upregulation of PI3K pathway^46^, thus highlighting the complex crosstalk between the different molecular pathways. Therefore, induction of PI3K-p85 seen in cases where resistance to afatinib and crizotinib combination treatment has developed could be inhibited by addition of a PI3K inhibitor.

In conclusion, we demonstrate that afatinib and crizotinib in combination is able to down-regulate key pathways like mTOR/Insulin through downstream effectors of the mTOR pathway, RPS6KB1 and RPS6, together with IRS-1 in the insulin signaling pathway. Furthermore, concomitant silencing of RPS6 and IRS-1 leads to further induction of cell death. Therefore, co-targeting proteins in both pathways might prove to be an attractive therapy option for CMM patients. Further validation studies are thus warranted to corroborate the impact of co-silencing insulin and mTOR signaling pathways as a potential clinical therapeutic regime.

## Supplementary information

Supplementary material and methods

Supplementary figure legends

Supplementary Figure S1

Supplementary Figure S2

Supplementary Figure S3

Supplementary Figure S4

Supplementary Figure S5

Supplementary Figure S6

Supplementary Figure S7

Supplementary Figure S8

Supplementary table legends

Supplementary table

RPPA raw data file
