# Supplementary material for: Inhibiting insulin and mTOR signaling by afatinib and crizotinib combination fosters broad cytotoxic effects in cutaneous malignant melanoma
Source: Cell Death Dis. 2020 Oct 20;11(10):882. doi: 10.1038/s41419-020-03097-2 (PMC7576205; doi:10.1038/s41419-020-03097-2)
Supplement: Supplementary file 1 — Supplementary material and methods [file 41419_2020_3097_MOESM1_ESM.docx]

**Supplementary material and methods**

**Clinical samples**

Clinical samples were collected as FFPE sections from 10 CMM patients with stage III/IV disease and as fresh frozen core biopsies from 2 matched stage IV cases (taken before treatment and at progression) of patients with *BRAF* mutant tumors treated with BRAFi. Tissue microarray (TMA) was purchased from US Biomax (ME 1002A). For details of samples, see manufacturer’s website (https://www.biomax.us/tissue-arrays/Melanoma/ME1002a). Additionally, TMA containing 65 tumor samples in duplicates from 56 patients with advanced melanoma (Stage III/IV) were generously donated by Prof. Meenhard Herlyn at the Wistar Institute, USA. This study has been approved by the regional ethics committee in Stockholm, Sweden (Dnr 2006/1373-31/3, Dnr 2011/1980-31/1, Dnr 2016/1506-32, Dnr 2017/947-32), the Institutional Review Board (IRB) (2802240) and has been conducted in accordance with the ethical principles given in the Helsinki Declaration. Informed consent was obtained from all patients included.

**Cell lines**

A375 was purchased from American Type Culture Collection (ATCC); A375VR4 was induced vemurafenib resistant subline of A375 as previously described ^1^. SkMel2, ESTDAB102 and cell lines ESTDAB105 were obtained from European Searchable Tumor Line Database and Cell Bank (ESTDAB). 1205-Lu, 1346 and 3918 were obtained as gifts from Prof. Meenhard Herlyn, Wistar Institute, USA. Cells were cultured in either MEM (supplemented with 1% sodium pyruvate and 1% non-essential amino acids) or RPMI-1640 media (Thermo Fischer Scientific). 10% FBS and 1% Pe-St were added to both media. All cell lines were confirmed to be mycoplasma free using LookOut Mycoplasma PCR detection kit (Sigma Aldrich, Stockholm, Sweden) and were used for experiments between passages P2-P12. Normal human keratinocytes and adult human dermal fibroblasts were obtained from Thermo Fischer Scientific. Normal human epithelial keratinocytes were cultured in Epilife media (Thermo Fischer Scientific) supplemented with 1% HKGS and 1% Pe-St. Human dermal fibroblasts, adult were cultured in Media 106 (Thermo Fischer Scientific) supplemented with 1% LSGS and 1% Pe-St. Keratinocytes and fibroblasts were used for experiments between passages P2-P4.

**Generation of resistant cell lines**

A375 cells were plated in T25 flasks at 80% confluency. Following day, cells were treated with IC50 drug concentrations of afatinib, crizotinib or the combination. Cells were checked regularly and dying cells were washed away with PBS and media containing drugs was replenished every 2-3 days. Surviving cells were re-plated in T75 flasks and cultured with media containing the drugs for 2 months. Resistant cells were verified with IC50 values.

**siRNA Transfection**

siRNA sequences (Dharmacon) were used to knockdown IRS-1 and RPS6 (sequences can be found in supplementary table 4). Non-targeting negative control siRNA (Dharmacon) was used control. All siRNAs were transfected using Lipofectamine 2000 (Sigma-Aldrich Chemie Gmbh, Munich, Germany) according to manufacturer’s recommendations.

**Immunofluorescence**

Immunofluorescence was performed using TMA and FFPE samples as previously described on manufacturer’s website (Novus Biologicals, USA). In brief, sections were dehydrated and antigen retrieval was performed using citrate buffer followed by over-night staining with antibodies listed in Supplementary Table 3. Next day, sections were washed, incubated with secondary antibodies (1:200, rabbit Alexa Flor 488 or mouse Alexa Flor 594, Cell Signaling Technologies, USA), mounted with DAPI (Sigma Aldrich), visualized and imaged using AxioImager M2 (Zeiss). For estimating % staining intensity, the images were taken at 10X magnification and the entire tissue section was considered during the evaluation process.

**Cell proliferation assay**

In 96 well flat bottomed plates, approximately 4000 cells/well were plated overnight. The following day cells were exposed to either afatinib (2µM) or crizotinib (2µM) (Sellekchem, USA) alone or in combination for 72 h followed by addition of MTS solution (Promega, Madison, WI, USA) and absorbance measurement at 490 nM using Tecan Spark 10M plate reader (Tecan Trading AG, Switzerland) to determine the inhibitory concentration of the drugs according to manufacturer’s protocol.

**Colony formation**

1000 cells/well were plated in 6 well plates overnight. Cells were transfected with siRNAs against IRS-1 (#3, #4) and RPS6 (#1, #3) in single or combination for 48h after which media was replaced with regular media. Colonies were allowed to form for an additional 10 days, with the media being replaced every 2-3 days. Cells were fixed for 20 min using 4% buffered formaldehyde. Colonies were stained with 0.05% crystal violet solution for 10 min following two washes with 1X PBS. Stained plates were scanned. To estimate amount of colony formation, crystal violet was dissolved in 100% methanol. In a 96 well plate, the crystal violet was diluted in 1:10 and absorbance was measured at 540 nm using Tecan Spark 10M plate reader instrument.

**Flow cytometry**

For FACS analysis, 3000 cells/well were plated in 12 well plates overnight. Following day, cells were transfected with IRS-1 # 3, #4 and RPS6 #1, #4 siRNA (single or with both siRNAs) for 48h. Cells were then trypsinized collected and spun down. Pellets were washed once with 1X PBS and then stained for 10 minutes in the dark on ice with 2% Annexin V and 2% PI solution (Sigma-Aldrich Chemie Gmbh, Munich, Germany). Additional 200 µL FACS incubation buffer was added after incubation and analysis was performed using Novocyte 3000 and Novoexpress software (ACEA Biosciences, San Diego, CA, USA) to determine induction of apoptosis and necrosis.

**Cell lysis for western blot and RPPA**

Whole lysates from cell lines was extracted in RIPA lysis buffer containing phosphatase and protease inhibitors as previously described in ^2^. For tumor tissue from xenografts, 50mg of tissue was homogenized and processed in lysis buffer as per recommendation by the MD Anderson Cancer Center Functional Proteomics Core Facility. Protein lysates were denatured using standard methods, measured using BCA reagent (Thermo Fischer Scientific, Germany) and used for western blot analysis or for RPPA analysis conducted at the MDACC Functional Proteomics Core Facility. The data was analysed as previously described in ^3, 4^. Raw data associated with the RPPA studies can be found within the supplementary information files.

**Cell Lysis for proteomic analysis**

Harvested cell pellets were resuspended in lysis buffer supplemented with phosphatase inhibitor (Roche Applied Science, Switzerland). Cells were sonicated and centrifuged and protein concentration was determined as mentioned before using BCA reagent kit (Pierce, Sweden). The protein lysates were reduced, enzymatically digested and the proteome and phosphoproteome was analysed using a label free method.

**Phosphoproteomics analysis**

200μg of tryptic digest of each sample underwent TiO_2_ phosphopeptide enrichment step as previously described ^5^; and analysed by LC-MS/MS using a Fusion OrbiTrap (Thermo Fisher, Germany). The mass spectrometer was connected to a Dionex UHPLC system (Thermo Fisher Scientific, Germany).

**LC-MS/MS analyses**

The proteome and phosphoproteome of each sample were analyzed on a Fusion Orbitrap (Thermo Fisher, Germany) as previously described in ^5^.

**Proteomic and phosphoproteomic data analyses**

The raw data were analyzed using MaxQuant 1.5.3.30 ^6^ and Andromeda ^7^ was used to search the MS/MS data against the UniProt *Homo sapiens* database (containing canonical and isoforms_42259 entries downloaded on 15^th^ January 2018) complemented with a list of common contaminants, and concatenated with the reversed version of all sequences as previously described in ^5^. Data analysis was executed using Perseus (1.6.1.2). Only protein/phosphopeptides with expression levels >1.5-fold changes (p<0.05) were considered for further analyses. The mass spectrometry proteomics data have been deposited to the ProteomeXchange Consortium via the PRIDE. Data are available with identifier PXD016788.

**Statistical analyses**

All experiments were performed in duplicates or triplicate and representative results were presented where data was expressed as mean ± SD or mean± SEM as mentioned in Figure legends. For analyzing the RPPA data, one way analysis of variance (ANOVA) was used to assess the differences in protein expressions between cell lines on a feature-by-feature basis. First, for one feature (protein) at a time, we carried out an over-all F test to detect any significant difference among the means of all the groups. Next, for the featured (proteins) identified in this process, we compared between desired cell line groups to identify the sources of difference. The R library “multcomp” was used for this purpose. Furthermore, to account for multiple testing, we estimated the false discovery rates (FDR) of the overall test of the model using the Benjamini-Hochberg method. Statistical analysis of the MS data was done using Microsoft Excel. All remaining statistical analyses were carried out using GraphPad Prism v.7.0 or v.8.0 (GraphPad Software, La Jolla, CA, USA). For comparisons between patient groups, Mann-Whitney U-Test was used. For all other comparisons, Student’s t test was used.

**References**

1. Azimi A, Tuominen R, Costa Svedman F, Caramuta S, Pernemalm M, Frostvik Stolt M*, et al.* Silencing FLI or targeting CD13/ANPEP lead to dephosphorylation of EPHA2, a mediator of BRAF inhibitor resistance, and induce growth arrest or apoptosis in melanoma cells. *Cell Death Dis* 2017, **8**(8)**:** e3029.

2. Das I, Wilhelm M, Hoiom V, Franco Marquez R, Costa Svedman F, Hansson J*, et al.* Combining ERBB family and MET inhibitors is an effective therapeutic strategy in cutaneous malignant melanoma independent of BRAF/NRAS mutation status. *Cell Death Dis* 2019, **10**(9)**:** 663.

3. Davies MA, Stemke-Hale K, Lin E, Tellez C, Deng W, Gopal YN*, et al.* Integrated Molecular and Clinical Analysis of AKT Activation in Metastatic Melanoma. *Clin Cancer Res* 2009, **15**(24)**:** 7538-7546.

4. Gopal YN, Deng W, Woodman SE, Komurov K, Ram P, Smith PD*, et al.* Basal and treatment-induced activation of AKT mediates resistance to cell death by AZD6244 (ARRY-142886) in Braf-mutant human cutaneous melanoma cells. *Cancer Res* 2010, **70**(21)**:** 8736-8747.

5. Azimi A, Caramuta S, Seashore-Ludlow B, Bostrom J, Robinson JL, Edfors F*, et al.* Targeting CDK2 overcomes melanoma resistance against BRAF and Hsp90 inhibitors. *Mol Syst Biol* 2018, **14**(3)**:** e7858.

6. Cox J, Mann M. MaxQuant enables high peptide identification rates, individualized p.p.b.-range mass accuracies and proteome-wide protein quantification. *Nat Biotechnol* 2008, **26**(12)**:** 1367-1372.

7. Cox J, Neuhauser N, Michalski A, Scheltema RA, Olsen JV, Mann M. Andromeda: a peptide search engine integrated into the MaxQuant environment. *J Proteome Res* 2011, **10**(4)**:** 1794-1805.
