# Supplementary material for: Inhibiting insulin and mTOR signaling by afatinib and crizotinib combination fosters broad cytotoxic effects in cutaneous malignant melanoma
Source: Cell Death Dis. 2020 Oct 20;11(10):882. doi: 10.1038/s41419-020-03097-2 (PMC7576205; doi:10.1038/s41419-020-03097-2)
Supplement: Supplementary file 2 — Supplementary figure legends [file 41419_2020_3097_MOESM2_ESM.docx]

**Supplementary Figure S1:** 2D MTS assay showing changes in cell viability for (a) three CMM cell lines (1205-Lu, 1346 and 3918) and (b) in two normal cells- keratinocytes and fibroblasts included in this study after treatment with afatinib (2µM), crizotinib (2µM) or the combination for 72h (error bars represents mean ± SD; n=3; p<0.0001, Student’s t test).

**Supplementary Figure S2:** (a) PCA analysis of whole proteomics and phospho-proteomics MS data. (b) Venn-diagram generated by using Venny 2.0 showing number and percentages of significantly altered proteins (p<0.05) using pairwise comparisons (combo vs DMSO, combo vs afatinib and combo vs crizotinib) for A375 and SKMel2.

**Supplementary Figure S3:** Altered GO biological processes taking into account the top 100 up-regulated or down-regulated proteins for both phospho and whole proteomics in A375 and SkMEl2.

**Supplementary Figure S4:** Table showing the significantly (p<0.05) altered proteins as assessed by RPPA after 3h or 24h of treatment with afatinib or crizotinib alone or in combination. Listed candidates have been selected after comparing combo vs DMSO, combo vs afatinib and combo vs crizotinib where the candidates must occur in 2 out of 3 comparison arms.

**Supplementary Figure S5:** (a) Table showing the significantly (p<0.05) altered proteins in afatinib+ crizotinib combination arm compared to vehicle or single treatments from RPPA results on xenograft tumors. (b)Quantification of p-RPS6KB1 positive cells in xenograft tumors treated with vehicle, afatinib or crizotinib as single treatments or combination of the two (error bars represent mean ± SD; n=3; p<0.001, Student’s t test).

**Supplementary Figure S6:** Representative images as examples from TMA containing tumor samples from stage III/IV (n=65) showing (a,b), cytoplasmic+ membrane expression of IRS-1 and cytoplasmic+ nuclear expression of RPS6KB1, (c) nuclear expression of IRS-1 and cytoplasmic expression of RPS6KB1 and (d) islands of tumor cells expressing predominantly nuclear IRS-1 and RPS6KB1. (e) Close up view of whole tissue staining pattern of IRS-1(predominantly membrane localization) and RPS6KB1 (cytoplasmic + nuclear localization) also shown in Fig. 6c, top row, panel 3.

**Supplementary Figure S7:** mRNA and protein expression of IRS-1, RPS6KB1 and RPS6 based on *BRAF /NRAS* mutation status using data from TCGA (n=337) (p<0.01).

**Supplementary Figure S8: (**a) IC50 values of A375 cells sensitive and resistant to afatinib or crizotinib. (b)Table showing significantly altered candidates on comparing combo resistant (ComboR) vs sensitive or single resistant cell lines and single resistant (AfaR or CrizR) vs sensitive cell lines. (c) Ratio of PMEL, PI3K-p85-α, PI3K-p85-β, CD171, mTOR, GAB2, JAK2, PI3K-p110-α, PI3K-p110-β gene expression at progression vs pretreatment in a selected subset of patients (n=10) where sampling was done subcutaneously. * vemurafenib treatment; ** dabrafenib treatment. Data set used (Rizos, Menzies *et al*. 2014)
