# Supplementary figures and images for: Inhibiting insulin and mTOR signaling by afatinib and crizotinib combination fosters broad cytotoxic effects in cutaneous malignant melanoma
Source: Cell Death Dis. 2020 Oct 20;11(10):882. doi: 10.1038/s41419-020-03097-2 (PMC7576205; doi:10.1038/s41419-020-03097-2)

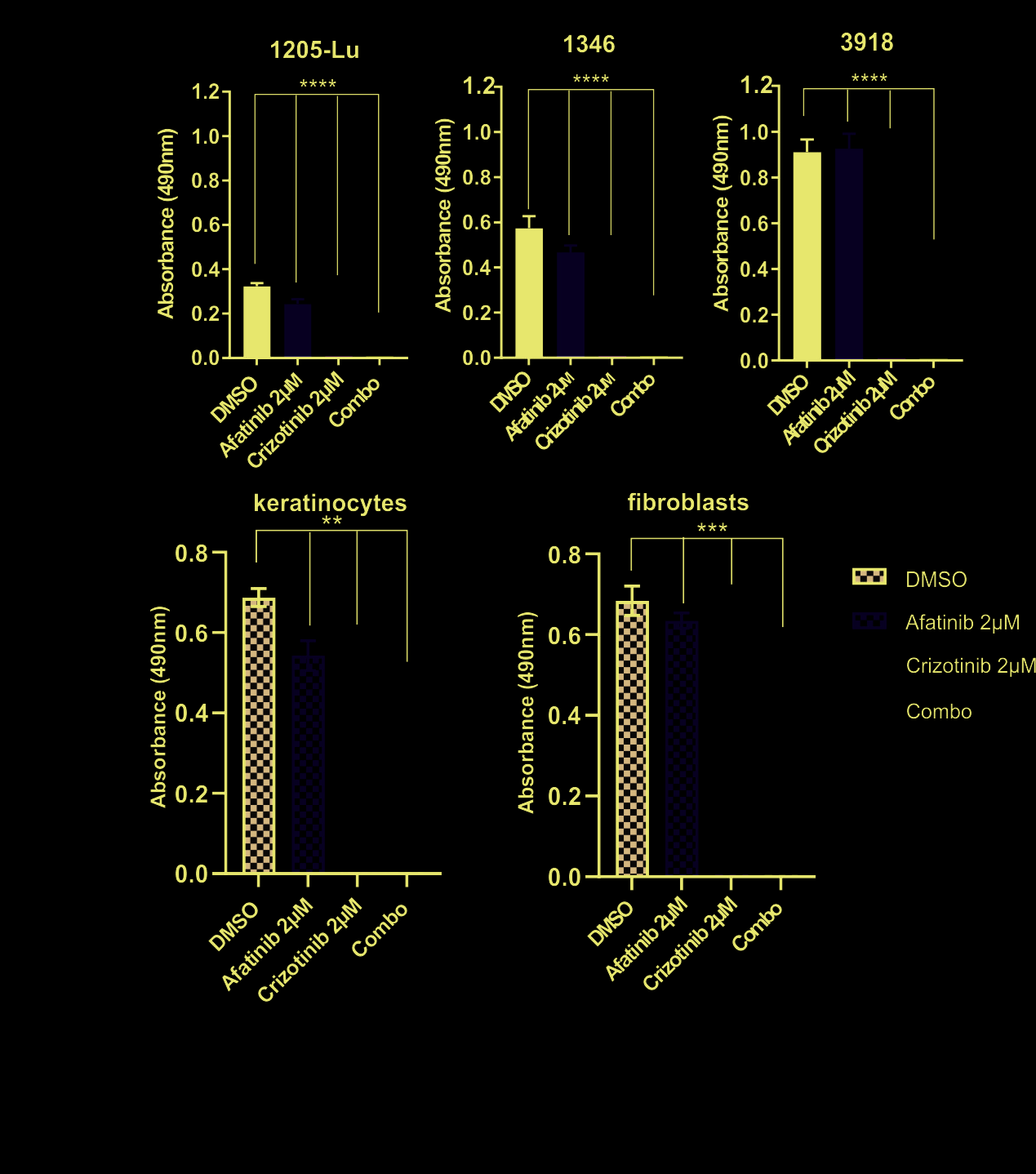

Supplement: Supplementary file 3 — Supplementary Figure S1 [file 41419_2020_3097_MOESM3_ESM.tif]

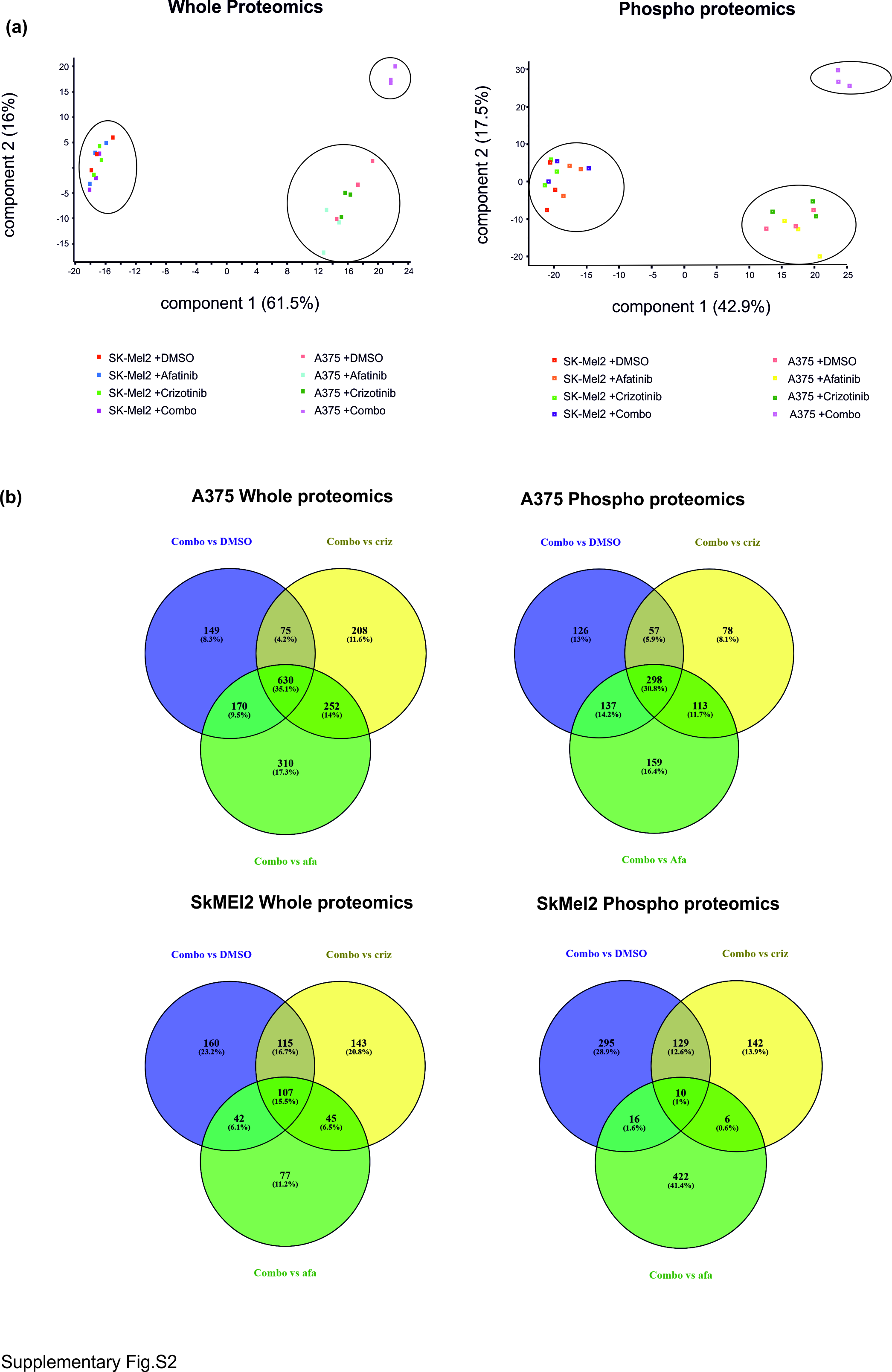

Supplement: Supplementary file 4 — Supplementary Figure S2 [file 41419_2020_3097_MOESM4_ESM.tif]

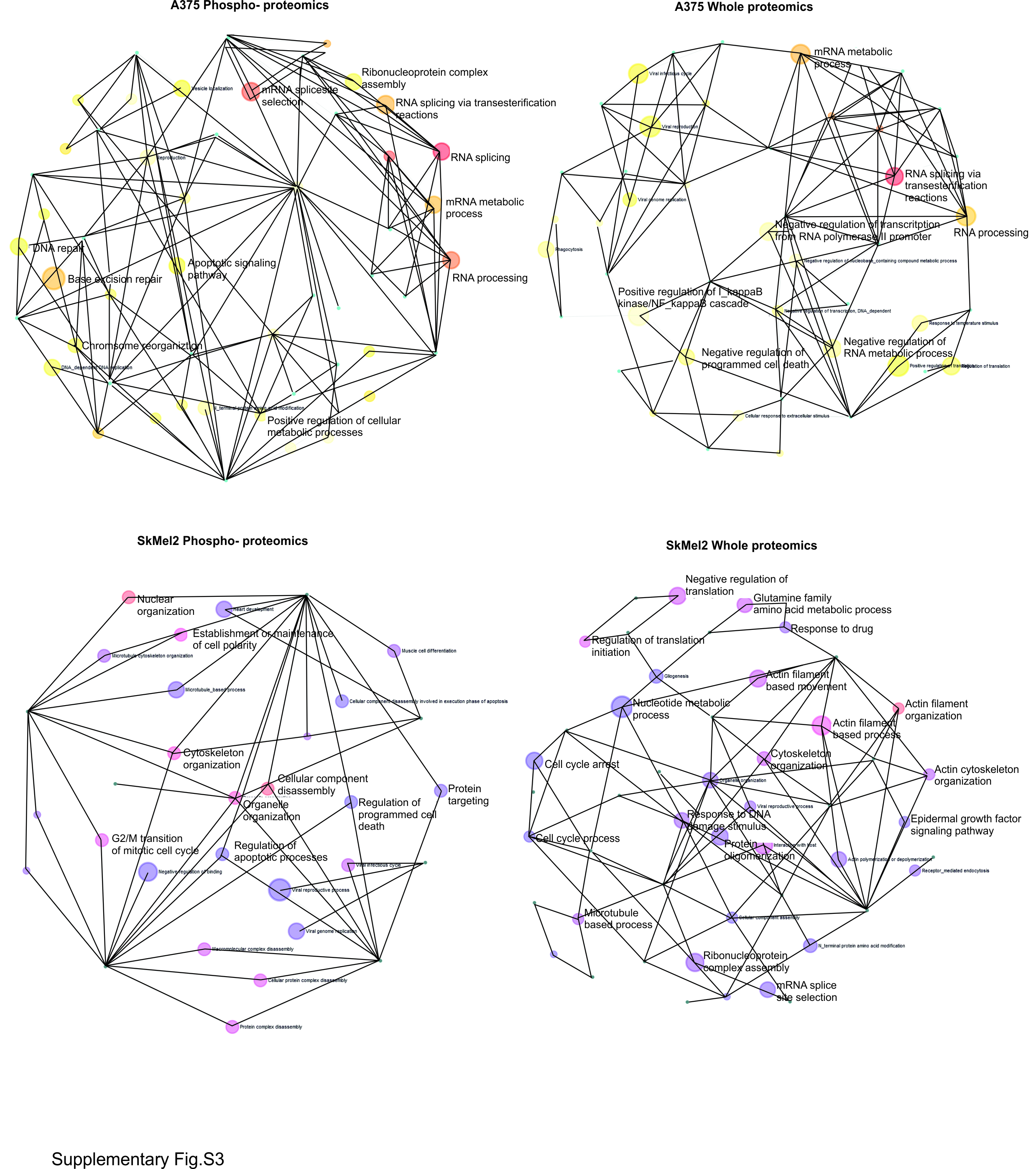

Supplement: Supplementary file 5 — Supplementary Figure S3 [file 41419_2020_3097_MOESM5_ESM.tif]

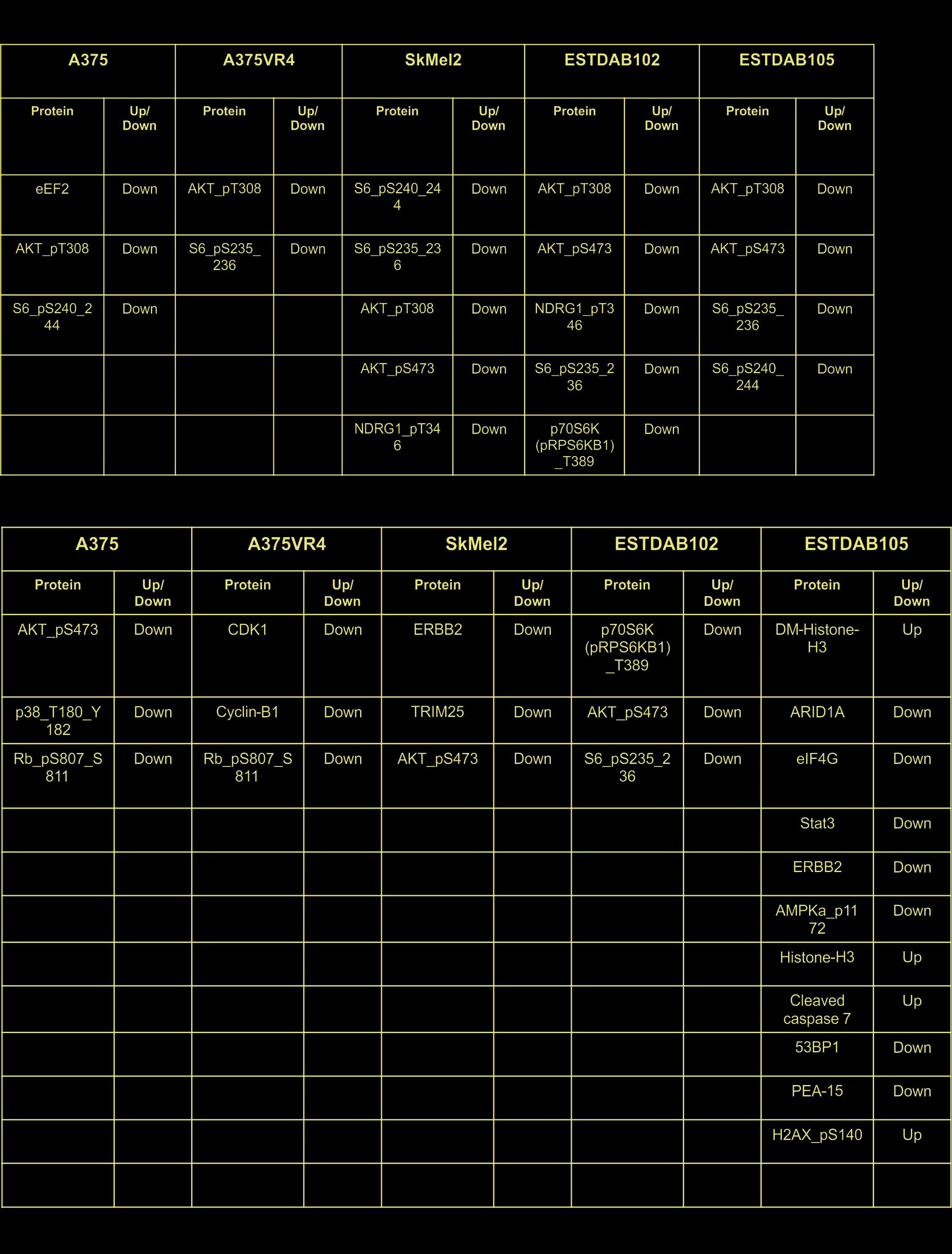

Supplement: Supplementary file 6 — Supplementary Figure S4 [file 41419_2020_3097_MOESM6_ESM.tif]

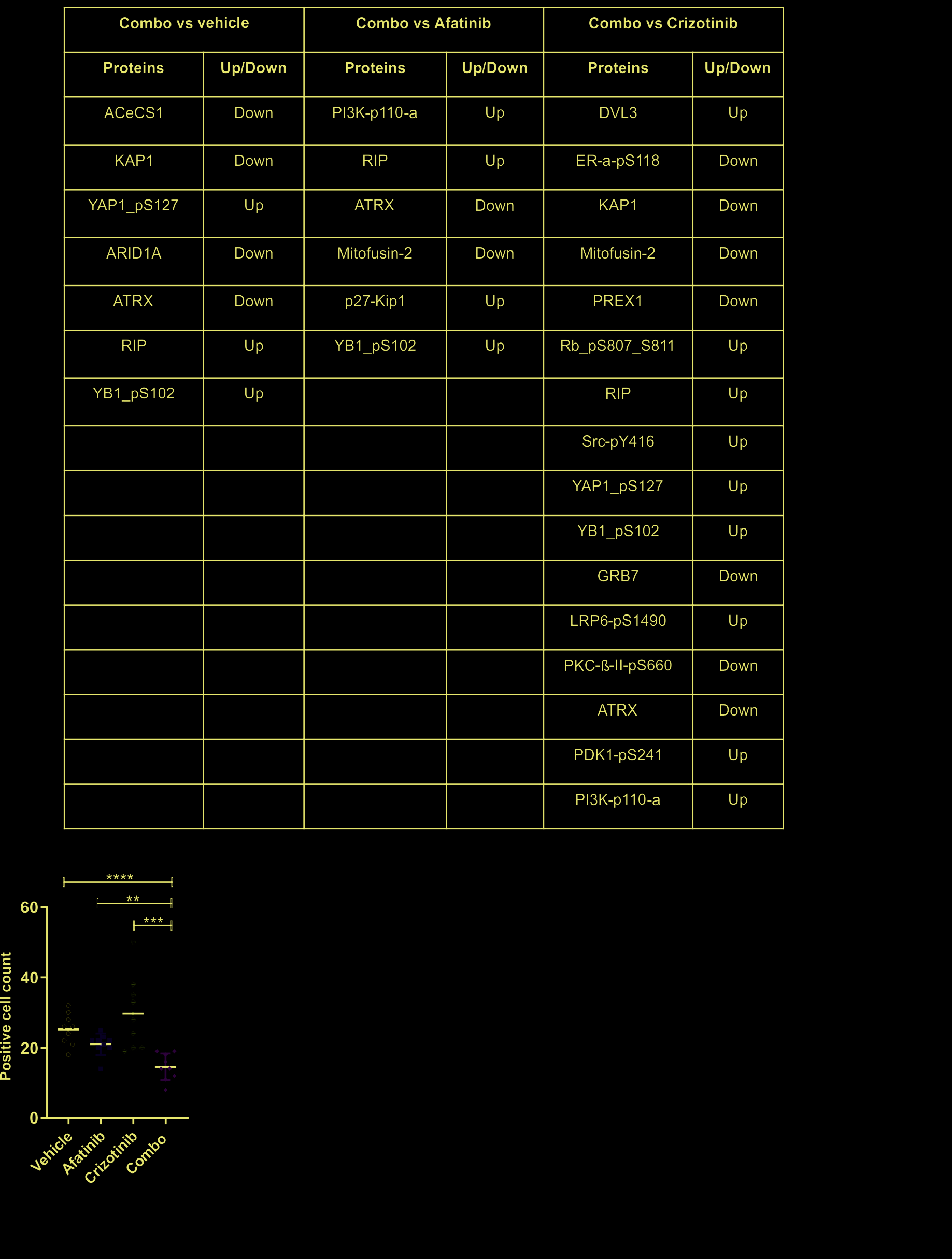

Supplement: Supplementary file 7 — Supplementary Figure S5 [file 41419_2020_3097_MOESM7_ESM.tif]

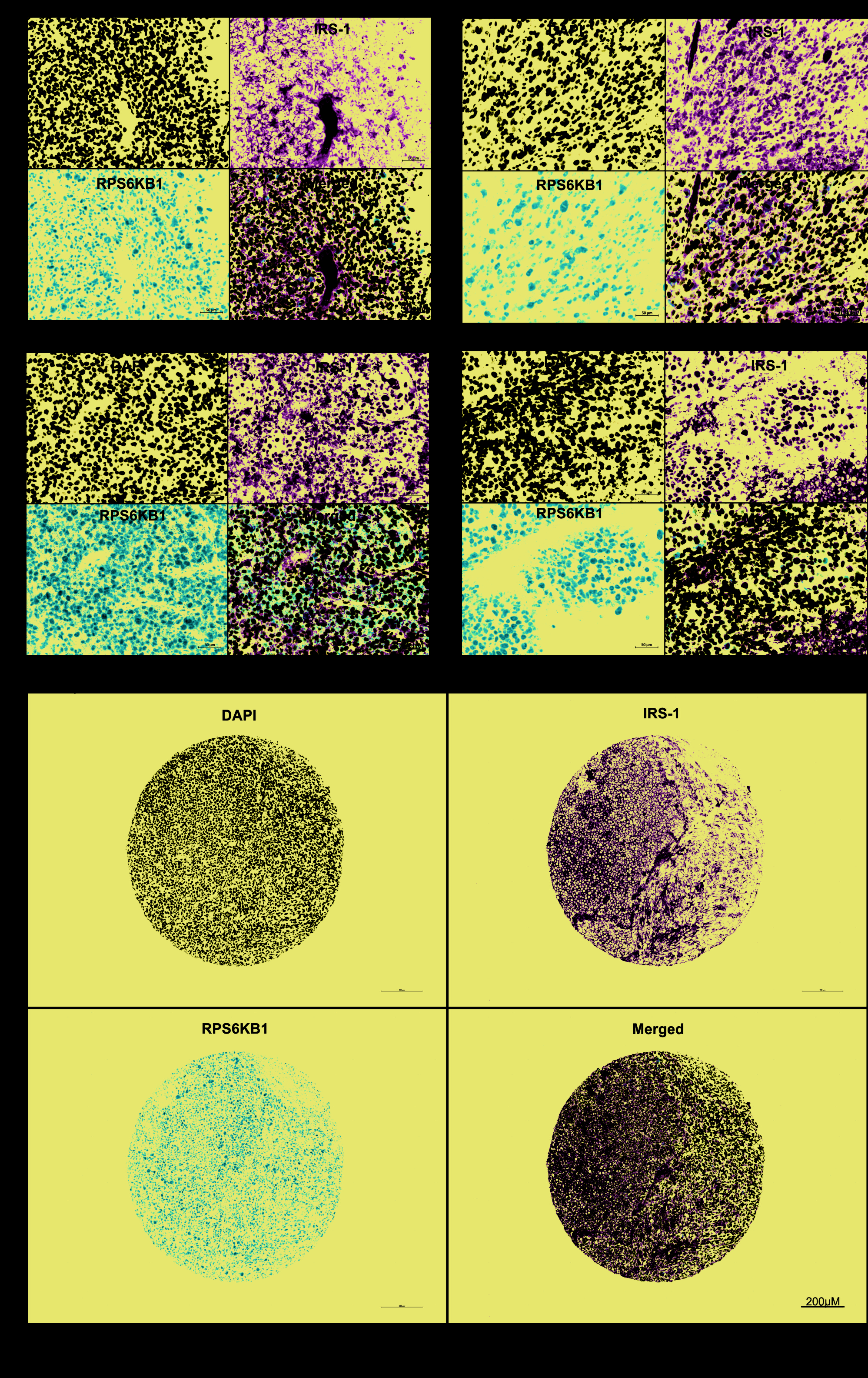

Supplement: Supplementary file 8 — Supplementary Figure S6 [file 41419_2020_3097_MOESM8_ESM.tif]

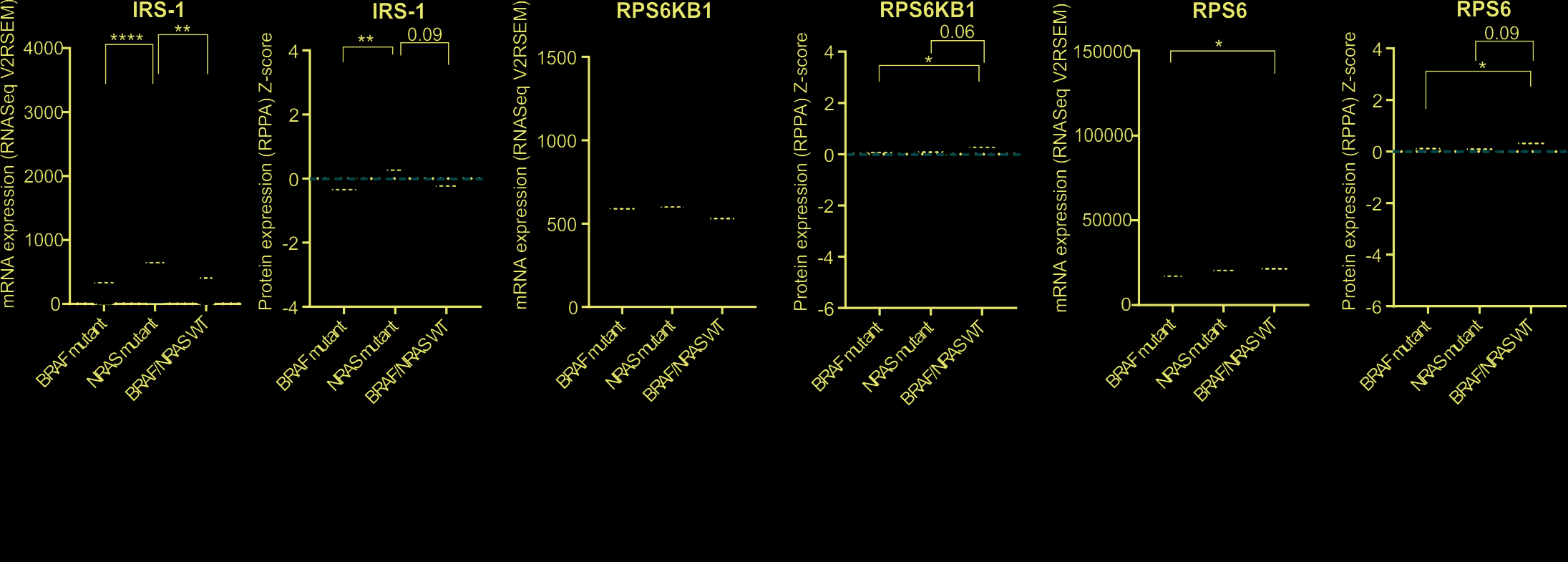

Supplement: Supplementary file 9 — Supplementary Figure S7 [file 41419_2020_3097_MOESM9_ESM.tif]

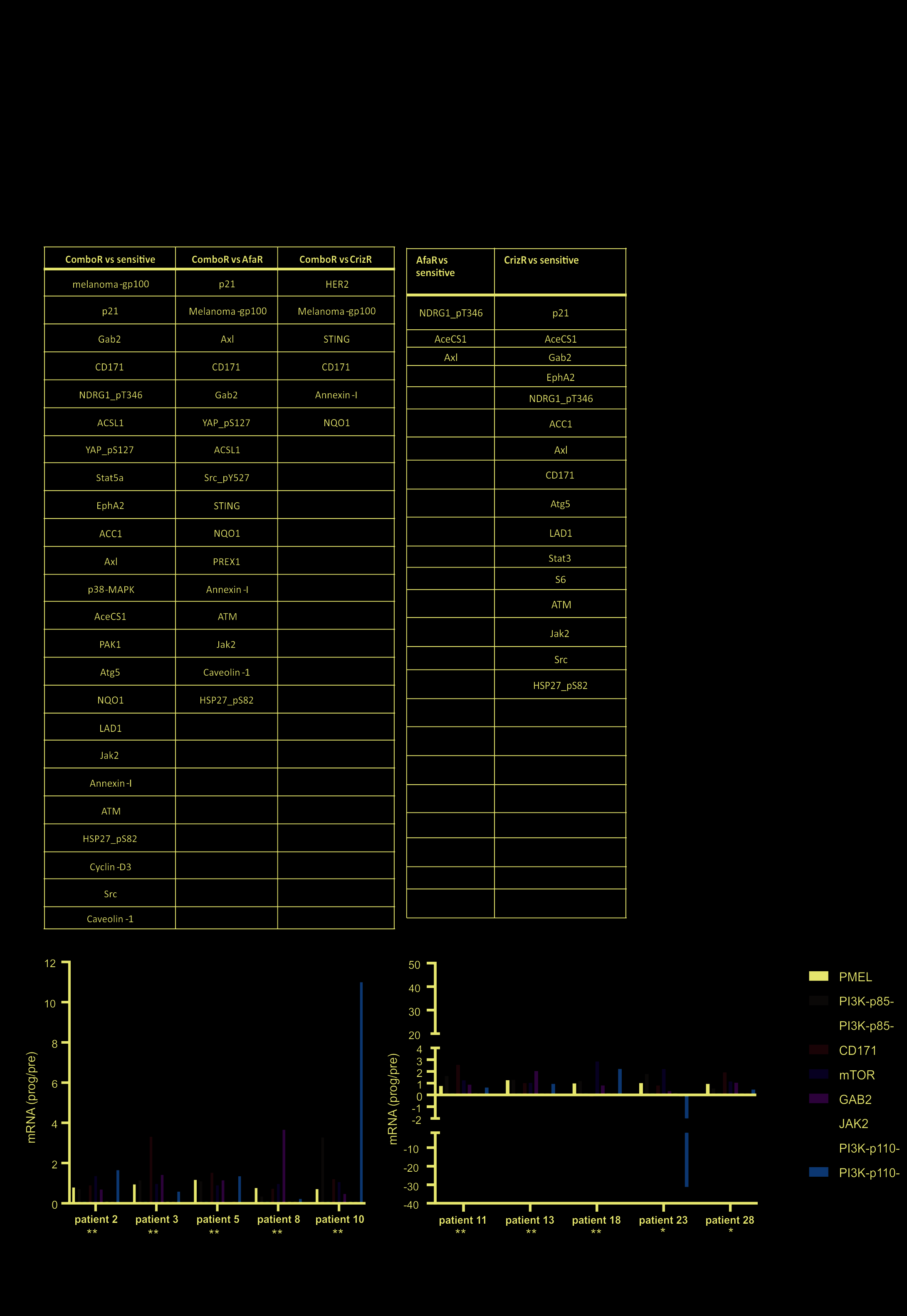

Supplement: Supplementary file 10 — Supplementary Figure S8 [file 41419_2020_3097_MOESM10_ESM.tif]
