# Supplementary material for: Inhibiting insulin and mTOR signaling by afatinib and crizotinib combination fosters broad cytotoxic effects in cutaneous malignant melanoma
Source: Cell Death Dis. 2020 Oct 20;11(10):882. doi: 10.1038/s41419-020-03097-2 (PMC7576205; doi:10.1038/s41419-020-03097-2)
Supplement: Supplementary file 11 — Supplementary table legends [file 41419_2020_3097_MOESM11_ESM.docx]

**Supplementary table legends:**

**Supplementary Table 1**

(a) KEGG pathway analysis of top 200 proteins up and down-regulated in A375 whole proteomic analysis). (b) KEGG pathway analysis of top 200 proteins up and down -regulated in A375 phospho- proteomic analysis. (c) KEGG pathway analysis of top 200 proteins deregulated in SkMel2 whole proteomic analysis). (d) KEGG pathway analysis of top 200 proteins deregulated in SkMel2 phospho-proteomic analysis).

**Supplementary Table 2**

Patient characteristics and corresponding staining and localization of IRS-1 and RPS6KB1 that have been included in Figure 3.

**Supplementary Table 3**

Table showing list of antibodies, the catalog numbers and supplier used in this study

**Supplementary Table 4**

Table showing siRNA sequences used against IRS-1 and RPS6. siRNA have been purchased from Dharmacon.
