# Supplementary material for: Inhibiting insulin and mTOR signaling by afatinib and crizotinib combination fosters broad cytotoxic effects in cutaneous malignant melanoma
Source: Cell Death Dis. 2020 Oct 20;11(10):882. doi: 10.1038/s41419-020-03097-2 (PMC7576205; doi:10.1038/s41419-020-03097-2)
Supplement: Supplementary file 12 — Supplementary table [file 41419_2020_3097_MOESM12_ESM.doc]

**Supplementary Table 1**a

| **Pathway** | **P.Value** |
| --- | --- |
| Spliceosome | 0,00022 |
| Non-alcoholic fatty liver disease (NAFLD) | 0,0046 |
| Huntington's disease | 0,0095 |
| PPAR signaling pathway | 0,012 |
| Cardiac muscle contraction | 0,014 |
| Oxidative phosphorylation | 0,038 |
| Parkinson's disease | 0,042 |
| mTOR signaling pathway | 0,048 |

**Supplementary Table 1**b

| **Pathway** | **P.Value** |
| --- | --- |
| Nucleotide excision repair | 0,0019 |
| Protein processing in endoplasmic reticulum | 0,022 |
| Huntington's disease | 0,029 |
| Mismatch repair | 0,032 |
| Citrate cycle (TCA cycle) | 0,042 |
| Base excision repair | 0,046 |
| DNA replication | 0,05 |

**Supplementary Table 1**c

| **Pathway** | **P.Value** |
| --- | --- |
| Valine, isoluecine and leucine biosynthesis | 0,0082 |
| Renal cell carcinoma | 0,0087 |
| Prostate cancer | 0,017 |
| MicroRNAs in cancer | 0,022 |
| Non-homologous end-joining | 0,027 |
| FoxO signaling pathway | 0,030 |
| 2-Oxocarboxylic acid metabolism | 0,037 |
| Pantothenate and CoA biosynthesis | 0,039 |
| Jak-STAT signaling pathway | 0,043 |
| Hepatitis B | 0,044 |
| RNA transport | 0,045 |
| Mismatch repair | 0,047 |

**Supplementary Table 1**d

| **Pathway** | **P.Value** |
| --- | --- |
| Arrhythmogenic right ventricular cardiomyopathy (ARVC) | 0,019 |
| EGFR tyrosine kinase inhibitor resistance | 0,020 |
| Hypertrophic cardiomyopathy (HCM) | 0,022 |
| Dilated cardiomyopathy | 0,023 |
| HIF-1 signaling pathway | 0,026 |
| Apoptosis | 0,035 |
| Apelin signaling pathway | 0,035 |
| Insulin signaling pathway | 0,035 |
| Ribosome | 0,039 |
| mTOR signaling pathway | 0,039 |

| **cases**  **Supplementary table 2**  **Supplementary Table 2** | **AJCC Stage at Bx** | **targeted therapy prior to Bx** | **Immune therapy prior to Bx** | **Other therapy prior to Bx** | **targeted seq: BRAF hotspot/ RAS/ NF1/ KIT/ GNAQ/ GNA11/ WT** | **IRS-1 signal** | **Localization** | **RPS6KB1 signal** | **Localization** |
| --- | --- | --- | --- | --- | --- | --- | --- | --- | --- |
| **1** |  |  |  |  |  | strong | nuclear | strong | nuclear |
| **2** | IV | post vem, mixed response, PFS 11 months | None |  | BRAFV600E | moderate | membrane+nuclear | strong | nuclear |
| **3** | IV | None | None | bone radiaton June 15 | FAILED | strong | cytoplams+ nuclear | strong | nuclear+cytoplasm |
| **4** |  | Dab + Tram D7 on | None |  | FAILED | strong | nuclear+membrane | strong | predominantly nuclear; some cytoplamsmic staining |
| **5** | IV | None | Ipilimumab x 4 cycles, PD | XRT to R axilla | BRAFV600E | moderate | nuclear+ membrane | strong | nuclear |
| **6** | IV | None | ipi, 2-4, 15, CR |  | G466E, NF1 | moderate | nuclear+membrane+cytoplasm | strong | nuclear+cytoplasm |
| **7** | IV | None | Ipilimumab x 4 cycles (received 2 cycles before surgery on 11/12/2013), PD | None | BRAFV600E | moderate | membrane | low | nuclear; few cytoplasmic + cells |
| **8** |  | none | none | NA | poor coverage | moderate | nuclear; <10% membrane | strong | nuclear+ cytoplasm |
| **9** | IIIB | None | Adjuvant interferon alpha 2b, d/c due to side effects | None | BRAFV600E | moderate | nuclear+membrane | low | few nuclear+ cells |
| **10** | IIIC | None | Ipilimumab, no tumor response | Dartmouth Regimen: involving a combination of three drugs: carmustine, dacarbazine, and cisplatinin, PR | DNA available MP1and MP2 | low | membrane+ few nuclear | low | very few nuclear+ cells |
| **11** | III | None | None | None | NF1 Q236X | moderate; strong in 40% | nuclear+membrane | moderate | nuclear+cytoplasm |
| **12** | IV | None | None | None | BRAFV600E | moderate | predominantly nuclear; <20% membrane | moderate | nuclear+cytoplasm |
| **13** |  | No Rx yet | none |  | DNA available MP1 | moderate | predominantly nuclear; <10% membrane | moderate | nuclear+cytoplasm |
| **14** | IV | None | None | None | BRAFV600E | low (necrotic tissue) | nuclear+membrane | not detected |  |
| **15** | IV | GSK436+ GSK212 | on ipi, 3 cycles, CR, tu free (3/13) |  | BRAFV600E | moderate | nuclear+membrane | strong | nuclear+cytoplasm |
| **16** | IV | None | None | Temsirolimus + HCQ, SD | BRAFV600R | strong | nuclear+membrane | moderate | predominantly nuclear; some cytoplasmic staining |
| **17** | IIIC | None | None | None | BRAFV600E | moderate | nuclear | moderate (~60% cells stained) | nuclear+cytoplasm |
| **18** | IV | post PLX | none |  | submitted?? | moderate | nuclear with islands of membrane+ cells | moderate | nuclear+cytoplasm |
| **19** | IV | None | None | Temozolomide (adjuvant: 6/2007/11/2007) | BRAFV600E | not evaluable |  | not evaluable |  |
| **20** | IV | None | None | None | BRAFV600E | strong | predominantly membrane; some nuclear | moderate | nuclear+cytoplasm |
| **21** | IV | None | None | post radiation neck, CR 2012 | too low conc to submit | strong | nuclear+membrane+cytoplasm | strong | nuclear+cytoplasm |
| **22** | IV | None | Tremelimumab/CD40- received 1 dose of Treme and 1 dose of CP 870,893, d/c due to toxicity; Ipilimumab- received 2 doses, d/c due to toxicity | XRT to chest | NF1 | difficult to analyze (fatty tissue) |  |  |  |
| **23** | IIIB | None | None | None | BRAFV600K | low (few islands of +ve cells) | predominantly nuclear+ some cytoplasm | low | predominantly nuclear+ some cytoplasmic staining |
| **24** | IV | None | None | None | KRAS G12D | low | membrane+ cytoplasm | moderate | nuclear+cytoplasm |
| **25** | IV | post PLX | now on ipi (3/13), SD |  | BRAFV600E HOM | moderate; strong in 15% of tissue | membrane + ~50% cells have nuclear staining | moderate | nuclear+cytoplasm |
| **26** | IV | PLX4032, on study responding, one s.c. lesion 10% increase | none | NA | BRAFV600E | moderate | predominantly nuclear; some cytoplasm | moderate | nuclear+cytoplasm |
| **27** | IV | on vem 1 month, PR, 2 months PFS | None | None | BRAFV600K | strong | nuclear | moderate | nuclear+ some cytoplasmic staining |
| **28** | IV | None | None | Temozolomide x 6 cycles given in combination with Leukine (adjuvant) | WT | strong | nuclear | strong | nuclear+ some cytoplasmic staining |
| **29** | IV | None | None | None | failed, need more DNA to resubmit | moderate (clones of cells with strong staining) | nuclear+membrane; cytoplasmic staining in < 5% of tissue | moderate | nuclear+cytoplasm |
| **30** | IV | one lesion progressed, syst. better, PLX ongoing | None |  | BRAFV600K | not evaluable; too few cells |  |  |  |
| **31** | IIIC | None | Ipilimumab, no tumor response | Dartmouth Regimen: involving a combination of three drugs: carmustine, dacarbazine, and cisplatinin, PR | WT | low | predominantly membrane; few nuclear+ cells | low | nuclear |
| **32** | IV | None | None | CyberKnife (brain) | BRAFV600E | moderate | nuclear; some scattered cells with cytoplasmic staining | moderate | nuclear |
| **33** | IV | 1 week on vem | None | None | BRAFV600E | low-moderate | membrane and cytoplasm | low | nuclear |
| **34** |  | naïve | None |  | DNA available MP1 | necrotic tissue--- difficult to analyze |  |  |  |
| **35** |  | no therapy | None |  | WT | moderate with islands of strong nuclear and cytoplasmic +ve cells | nuclear+ cytoplasm | moderate | nuclear+ cytoplasm |
| **36** | IV | 8/15-9/15 DCC2071 (cMET TIE2 inhibitor), PD; 10/15-ongoing Dabrafenib + Trametinib + HCQ | ipi 10/14-1/15, PR; PD-1 4/15-6/15 PD |  | FAILED | low | predominantly cytoplasmic +; some nuclear staining | low | nuclear; few scattered cytoplasmic + cells |
| **37** | IV | none | none | NA | WT | strong | nuclear | moderate | nuclear with few scattered cytoplasmic + cells |
| **38** | IV | None | Ipilimumab x 4 cycles, Post, PD | None | NRASQ61R | low-moderate | nuclear | moderate | nuclear+cytoplasm |
| **39** | IV | 11/13-4/14 dab/tram SD; | 9/13-12/13 ipi; | 12/13 and 1/14 gamma knife; 5/14 whole brain radiation; | BRAFV600E | low | nuclear | low | nuclear |
| **40** | III | None | None | None | WT | strong | membrane and nuclear with few cytoplasmic + cells | moderate | nuclear+cytoplasm |
| **41** | IV | vemurafenib 12/15/11- 12/26/11 | IFNalpha may-sep 2011, ipi oct-dec 2014 | radiation l neck | BRAFV600E | moderate | nuclear | low | nuclear |
| **42** | III | None | None | None | NA | moderate | nuclear | low | nuclear |
| **43** | IV | 11/13-4/14 dab/tram SD; | 9/13-12/13 ipi; | 12/13 and 1/14 gamma knife; 5/14 whole brain radiation; | BRAFV600E | moderate | predominantly nuclear; < 10% cytoplasmic staining in tissue | moderate | nuclear+ cytoplasmic |
| **44** | IIIA | None | None | None | BRAFV600K | moderate | predominantly membrane; < 10% nuclear staining in tissue | strong | nuclear+cytoplasm |
| **45** | IV | None | on PD-1 (since 1/24/14) |  |  | moderate | nuclear+membrane+ cytoplasm | moderate | nuclear+ cytoplasm |
| **46** | IIIA | None | None | None | BRAFV600K | moderate | membrane; one clone of cells with nuclear+ staining | moderate | nuclear; <5% cytoplamsic staining |
| **47** |  | None | PD-1 6/15-ongoing |  | WT | moderate | nuclear | moderate-strong | nuclear+ cytoplasm |
| **48** | III | None | Ipilimumab x 4 cycles (received 1 cycle before surgery on 11/6/2013), N/A | None | NRASQ61H, NF1 | moderate | nuclear+ some cytoplasmic staining | low | nuclear+ cytoplasm |
| **49** | III | None | None | None | NRASQ61R | not evaluable; too few cells |  |  |  |
| **50** |  |  |  |  |  | low-moderate | nuclear+ cytoplasm | strong | nuclear + cytoplasm |
| **51** | IV | 3/14-ongoing dab/tram, response NA | 10/12-2/13 ipi, response NA, progression yes; 3/15-ongoing PD-1 | post radiation neck | NF1 | moderate (staining in 60% of cells) | predominantly membrane;some nuclear + cells | moderate | nuclear+ cytoplasm |
| **52** | IV | PLX, pre/on/post?? | none |  | BRAFV600E | moderate | predominantly nuclear; < 2% cytoplasmic staining | moderate | nuclear+ cytoplasm |
| **53** | IV | None | Ipilimumab x 3 cycles, PD, Anti-PD1 x 5 cycles, PD | XRT to L acetabulum; XRT to L1-S1 | WT | low | nuclear | low | nuclear+ cytoplasm |
| **54** | IV | None | Adjuvant Leukine, N/A | None | WT | low | cytoplasm | low | nuclear+ cytoplasm |
| **55** | III | None | None | None | BRAFV600E | moderate | nuclear | moderate | nuclear+ cytoplasm (some cells have strong cytoplasmic staining) |
| **56** |  | none | none | NA | failed, no more DNA to resubmit | low; islands of cells with strong stain | nuclear+ cytoplasm | low | cytoplasm |
| **57** | IV | None | Ipilimumab x 3 cycles (received 1 cycle before surgery on 2/21/2014), PD; XRT to spine (received 3/11 fxs before surgery on 2/21/2014) |  | WT | moderate | nuclear; <25% cytoplasmic staining | strong | nuclear+ cytoplasm |
| **58** | IV | None | Ipilimumab x 1 cycle, PR, disc tox; Anti-PD1 x 14 cycles, PD, PFS 6 months |  | BRAFV600E | moderate | nuclear+ membrane | moderate | nuclear+ cytoplasm |
| **59** |  | none | none | NA | KRAS G12D | low; with islands of cells with strong staining | nuclear; <20% cytoplasmic staining | moderate | nuclear+ cytoplasm |
| **60** |  | none | none | NA | NF1 | moderate-strong | nuclear; <10% membrane+ staining | low | nuclear+ cytoplasm |
| **61** | IV | dab from 5/27/2011-2/2012, PD, switched to combo in 2/2012, SD ongoing | None |  | BRAFV600K | moderate | nuclear | strong | nuclear+ cytoplasm |
| **62** | IIIC/IV | None | None | None | BRAFV600E | strong | nuclear+ cytoplasm | strong | nuclear+ cytoplasm |
| **63** | IV | None | post ipi SD, PFS 3 months | post radiation, | WT | strong | nuclear+ cytoplasm | moderate | nuclear+ cytoplasm |
| **64** |  | none | none | NA | BRAFV600E | moderate | predominantly nuclear with clones of cytoplasmic + cells | moderate | nuclear+ cytoplasm |
| **65** | IV | None | post ipi 2 months (2010) PR (near CR); post ipi 3 months (2012) PD; post anti CD137 6 months (2013) PD, |  | NRASQ61K | moderate | predominantly nuclear with clones of cytoplasmic + cells | moderate | nuclear+ cytoplasm |

Supplementary table 3:

| **Antibody** | **Company** | **Catalog #** |
| --- | --- | --- |
| p-mTOR S2448 | Cell Signaling Technologies | 5536 |
| Total mTOR(Immunoblotting) | Cell Signaling Technologies | 2983 |
| Total mTOR (IF) | R & D Systems | MAB15371 |
| Total RPS6KB1 (IF) | Sigma-Aldrich | **WH0006198M1** |
| Total RPS6KB1 (Immunoblotting) | Cell Signaling Technologies | 2708 |
| p-RPS6KB1 T389 (Immunoblotting) | Cell Signaling Technologies | 9205 |
| p-RPS6KB1 T389 (IF) | Thermo Fischer Scientific | 701064 |
| p-RPS6S240-244 | Cell Signaling Technologies | 5364 |
| p-RPS6S235-236 | Cell Signaling Technologies | 4858 |
| Total RPS6 | Cell Signaling Technologies | 2217 |
| PDGFβ | Sigma-Aldrich | [SAB4502149](https://www.sigmaaldrich.com/catalog/product/sigma/sab4502149?lang=en&region=SE) |
| pEGFR Y845 | Cell Signaling Technologies | 6963 |
| Total EGFR | Cell Signaling Technologies | 4267 |
| pERBB3 Y1289 | Cell Signaling Technologies | 4791 |
| Total ERBB3 | Cell Signaling Technologies | 12708 |
| Total AXL | Cell Signaling Technologies | 8661 |
| Total IGF1R | Cell Signaling Technologies | 9750 |
| pAKT S473 | Cell Signaling Technologies | 4060 |
| Total AKT | Cell Signaling Technologies | 4691 |
| pERK p44-42 | Cell Signaling Technologies | 4696 |
| Total ERK | Cell Signaling Technologies | 9102 |
| AceCS1 (Immunoblotting) | Cell Signaling Technologies | 3658 |
| AceCS1 (IF) | Santa Cruz Biotechnology | 377149 |
| Total IRS-1 | Novus Biologicals | 82001 |
| p-IRS1 S307 | Cell Signaling Technologies | 2381 |
| β-actin | Cell Signaling Technologies | 12262 |
| Anti-biotin | Cell Signaling Technologies | 7705 |
| Anti-rabbit | Cell Signaling Technologies | 7074 |
| Anti-mouse | Cell Signaling Technologies | 7076 |
| Anti-rat (IF) | Thermo Fischer Scientific | 31629 |
| Anti-goat (IF) | Thermo Fischer Scientific | A-11058 |
| Anti-rabbit (IF) | Cell Signaling Technologies | 4412 |
| Anti-mouse (IF) | Cell Signaling Technologies | 8890 |
| MET | R & D Systems | AF527 |

Supplementary table 4:

| IRS-1 #1 | UCAAAGAGGUCUGGCAAGU |
| --- | --- |
| IRS-1 #2 | GAGCUAAGCAACUAUAUCU |
| IRS-1 #3 | GAACCUGAUUGGUAUCUAC |
| IRS-1 #4 | CCACGGCGAUCUAGUGCUU |
| RPS6 #1 | GGACGAUGAACGCAAACUU |
| RPS6 #2 | UAAAGAAGAUGAUGUCCGC |
| RPS6 #3 | UGGACUGACUGAUACUACA |
| RPS6 #4 | GAUGCAAAUCUGAGCGUUC |
